# Mating portfolio and neutral mechanisms are primary causes of genet-ramet frequencies and spatial distributions in smooth cordgrass (*Spartina alterniflora*) along salt marsh tidal gradients

**DOI:** 10.3389/fgene.2026.1810782

**Published:** 2026-05-01

**Authors:** Jewel Tomasula, Seamus Caslin, Gina M. Wimp, Matthew B. Hamilton

**Affiliations:** Department of Biology, Georgetown University, Washington, DC, United States

**Keywords:** clonal reproduction, environmental gradient, genetic diversity, ramets per genet, salt marsh, spatial genetic variation

## Abstract

Partially clonal plants are common and often act as ecological foundation species. Patterns of clonal genotypes (genets) and their vegetative modules (ramets) in natural populations remain poorly documented. Knowledge gaps in ecological genetics include if environmental variation promotes or limits ramet production, and if random mechanisms or natural selection mainly shape variation in the frequency distributions and spatial organization of ramets and genets. We used genetic markers to measure genet and ramet patterns of *Spartina alterniflora*, a foundation plant species in North American Atlantic coastal salt marshes, along its natural gradient of tidal inundation and interspecific competition. In ten patches in a natural salt marsh, we sampled 935 *S. alterniflora* stems on 1 m grid transects spanning natural environmental gradients. Samples were genotyped with ten microsatellite loci that provided high exclusion probabilities. Genotypes showed 223 multilocus lineage genets (MLLs) genets that were unique to patches (except a single MLL) and excess homozygosity consistent with biparental mating among relatives or as much as 50% selfing. Patches showed low to no genetic differentiation and there was no isolation by distance within nor between patches. Distributions of ramets per genet were consistent with random sampling with replacement that was heterogeneous among patches, except three MLLs from two patches with high ramet counts. Spatial patterns of ramets within MLLs exhibited intermediate aggregation and interspersion consistent with vegetative expansion by rhizomes. Genotype frequencies, ramet and genet spatial patterns, and distributions of ramets per genet were overall consistent with life history (mating portfolio, vegetative growth by rhizomes) and neutral sampling mechanisms. The three MLLs with high ramet counts could be explained by a range of neutral mechanisms such as patch disturbance history or priority effects, while adaptation to environmental gradients of a few meters is unlikely since these MLLs spanned large areas. As ecosystems dominated by partially clonal species experience a myriad of anthropogenic impacts, our results support wider recognition of combined sexual and vegetative mating portfolios in foundation plant species and improved quantification of clonal patterns on landscapes to better understand and predict ecological genetic variation.

## Introduction

Spatial patterns of biodiversity are often shaped by environmental factors (Manel et al., 2003; Tilman et al., 2014). Habitat characteristics influence dispersal (“what can get where”) and survival (“what can live where”), and some habitats are closely associated with certain disturbances or stress gradients (Manel et al., 2003). However, neutral factors independent of environmental conditions can also affect these spatial patterns of biodiversity. Neutral theories of community assembly predict that biodiversity within a community is maintained by stochastic factors like birth, death, and dispersal rather than by functional fit to environmentally-determined niches (Hubbell, 2001). Determining the balance of factors that produce biodiversity is particularly important within foundation species, which play an outsized role in ecosystem functioning. Genetic diversity within populations of foundation species has been shown to influence trophic interactions and ecological processes (Whitham et al., 2003; Hughes et al., 2008; Raffard et al., 2018). Therefore, identifying how a foundation species’ genetic diversity varies within a population is critical to understanding the role that population plays in the broader ecosystem.

Many foundation species exhibit a degree of clonal spread, such as in terrestrial and aquatic grasses, riparian trees, and corals (Ellstrand and Roose, 1987; Barrett, 2015). In such clonal species, patterns of intraspecific genetic variation are the product of a mating portfolio, the combination of outcrossing, self-fertilization, and clonal growth (Barrett and Harder, 2017). Plant mating portfolios determine genotype frequencies and heterozygosity, and shape the spatial distribution of genotypes within populations. In clonal or vegetative growth, individuals of distinct genotypes (genets) produce somatic modules (ramets) that can remain physiologically connected or become disconnected, grow and expand or spread, and disperse (Harper, 1977; 1980). Ramets themselves are capable of independently reproducing both sexually and by further vegetative growth. Clonal reproduction may permit genets to expand in available habitat space quickly, but with potential costs such as intragenet mating (geitonogamy) that can lead to a reduction in pollen export or an increase in self-fertilization (Handel, 1985). The production, spread, and possible death of ramets over time, in combination with the anatomical structures that produce ramets (e.g., tillers, stolons, rhizomes, etc.), are expected to shape the spatial distributions of genets and ramets (Harper, 1980; Harada et al., 1997; Xiao et al., 2015). Possible spatial distributions range from “phalanx” genets where ramets are tightly grouped to “guerrilla” genets with their ramets widely spaced and intermixed with ramets of other genets (Harper, 1980; Doust, 1981; Vallejo-Marin et al., 2010; Barrett, 2015).

In addition to clonal reproductive strategies, environmental factors like disturbance may also promote or limit genet and ramet patterns in partially clonal populations. Physical disturbances may clear areas for recruitment and establishment, possibly altering diversity, but low and high disturbance frequencies or magnitudes may tend towards monoclonal populations as one genet outcompetes others (Wilkinson, 1999; Honnay and Bossuyt, 2005; McMahon et al., 2017). Stress gradients influence species diversity, particularly of sessile organisms such as plants and corals, and may similarly filter genets within populations that better survive or spread in higher stress conditions (Torres et al., 2020). Habitat edges, and the interspecific interactions associated with them, may also impact clonal spatial patterns. For example, ramets may better establish and compete with neighboring plants of other species compared to seed progeny. While prior studies have characterized genetic diversity in partially clonal foundation species (e.g. Blum et al., 2007; Madritch et al., 2009; Becheler et al., 2010; Torres et al., 2020), studies that explicitly consider how the environment, neutral mechanisms, and mating portfolios combine to influence genetic spatial patterns are still needed to reveal a complete picture of intraspecific genetic diversity in ecosystems dominated by partially clonal species.

The partially clonal salt marsh foundation species *Spartina alterniflora* (Loisel.) has been widely studied and provides a useful system for testing mechanisms shaping genotype variation and patterns of genets and their ramets. *Spartina alterniflora* occurs in temperate salt marshes from Atlantic Canada to the Gulf coast of the United States and Mexico, with disjunct populations in Argentina. Across much of its northern range, *S. alterniflora* occupies a tidal elevation gradient described as low marsh and high marsh (Bertness, 1991). At the low marsh end, plants occur adjacent to tidal creeks and experience frequent inundation, high salinity, and disturbance from wrack (Brewer et al., 1998) and, in northern portions of the range, winter ice shearing (Ewanchuk and Bertness, 2003). At high marsh end, this gradient terminates at a habitat boundary with *Spartina patens*, a less flood-tolerant species that can competitively exclude *S. alterniflora* (Bertness, 1991); the high marsh boundary is most characteristic of the mid-Atlantic and northern portion of the range where *S. patens* occurs. Across this natural environmental gradient, *S. alterniflora* exhibits phenotypic differentiation between low-marsh (tall-form) and high-marsh (short-form) plants (Valiela et al., 1978; Gallagher et al., 1988), driven by interacting environmental, genetic, and epigenetic processes (Mounger et al., 2022).

Previous work on *S. alterniflora* suggests that environmental stress across the tidal gradient may structure clonal spatial patterns and genetic diversity, but key mechanisms remain unresolved. Distinct tall-form phenotypes at tidal creekside and short-form phenotypes in the interior high marsh have led to the assumption of genetic divergence (Hughes and Lotterhos, 2014; Zerebecki et al., 2021; Hanley et al., 2025), although experimental evidence of whether there are genetic differences between tall- and short-form phenotypes has been mixed (Valiela et al., 1978; Gallagher et al., 1988). Observations of vegetative reproduction, which would influence clonal spatial pattern, indicate that tiller density peaks near the *S. patens* boundary (Bertness, 1991) and that stressors such as tidal inundation are associated with clonal growth (Hughes and Lotterhos, 2014). At the same time, disturbance has been proposed to promote genetic diversity and flowering in *S. alterniflora* (Edwards et al., 2005; Noto and Hughes, 2020; Walker et al., 2021; Zerebecki and Hughes, 2013; Li and Pennings, 2017). Some studies suggest that clonal structure may be dominated by a few well-adapted genets (Proffitt et al., 2003; Walker et al., 2021). However, prior genetic and clonal studies have important limitations: many either mapped clonal structure without incorporating environmental variation or alternative mechanisms (e.g. Walker et al., 2021), or quantified fine-scale genetic variation using sampling and models that assumed sexual reproduction and neglected clonal ramets (e.g. Hughes and Lotterhos, 2014). Other studies have focused on broader population-level patterns without resolving fine-scale spatial structure (Blum et al., 2007; Utomo et al., 2009; Novy et al., 2010). Together, these gaps limit our ability to distinguish among environmental filtering, disturbance, and neutral clonal processes in shaping intraspecific genetic diversity.

Here we report a study that employed genetic markers to identify clonal genets and ramets and their frequencies and spatial distributions over a tidal inundation gradient in a natural population of *S. alterniflora*. Using multilocus microsatellite genotype data, we quantified distinct genets and their vegetative ramets, the distributions of ramets per genet, and the spatial patterns of genets and ramets on the landscape. The main questions we addressed were: 1) what is the mating portfolio, or combination of outcrossing, self-fertilization, and vegetative growth, that produced the observed genotypes?; 2) what was genet richness, what were counts of ramets per genet, and did ramets per genet differ from a null model of random sampling with replacement?; and 3) were clonal genet and ramet spatial patterns coincident with the natural salt marsh environmental gradient? We employed explicit quantitative ecological neutral and population genetic models for hypothesis testing, an approach that has not been widely applied to *S. alterniflora* nor to clonal plants in ecological genetic studies. We found that patterns of genets and ramets in this *S. alterniflora* population were consistent with rhizomatous plant growth life history and neutral sampling mechanisms, and there was little evidence that genet and ramet patterns were shaped by adaptation to salt marsh tidal gradients.

## Materials and methods

### Species and study sites

We sampled *S. alterniflora* (also identified as *Sporobolus alterniflorus*), or smooth cordgrass, from one population in a natural salt marsh in the Mullica River Great Bay estuary in New Jersey, United States (39^0^ 31.6′N, 74^0^ 19.2′W) in summer 2018. *S. alterniflora* is a perennial grass that reproduces both sexually and asexually via rhizomes and tillers. *Spartina alterniflora* is wind pollinated and protogynous (female flowers mature before male flowers), and thought to be predominantly outcrossing (Bertness and Shumway, 1992; Daehler and Strong, 1994; Fang et al., 2004). The seeds of *S. alterniflora* are dispersed via hydrochory during tidal flooding and heavy rain events (Elsey-Quirk et al., 2009). *Spartina alterniflora* spans a gradient from the low marsh tidal creeks to the high marsh border with *S. patens*.

### Sample collection

**FIGURE 1:**
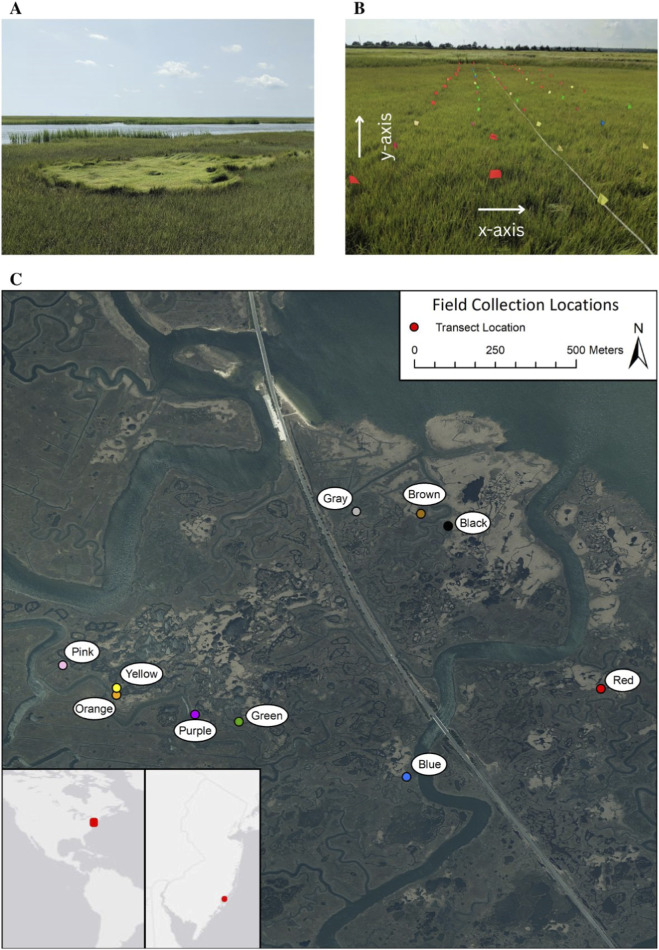
Images of the study site, transect sampling grid within one patch, and the geographic locations of sampled patches. **(A)** Example of the natural salt marsh gradient, where *Spartina alterniflora* (dark green) grows between a tidal creek and a patch of higher elevation *S. patens* (light green). **(B)** Example of the transect sampling grid within one patch where each flag marked a sampling point. **(C)** Map of ten patch locations at the Great Bay Boulevard Wildlife Management Area in New Jersey, United States, with insets showing the sampling location at the scale of coastal New Jersey, and North and South America.

Sampling locations were approximately 250 m to more than 1,000 m apart (Figure 1). We selected sampling locations where a *S. patens* patch was at least 5 m wide. At each sampling location, we set up grid transects across a *S. alterniflora* patch—from the *S. patens* edge to the tidal creek edge—in five parallel lines separated by 1 m with sample points at 1-m intervals (Figure 1). All patch sampling grids were oriented with transects (arbitrarily designated the x-coordinate) perpendicular to the tidal creeks and the distance along each transect (arbitrarily designated the y-coordinate) indicating how far along that axis a sample was taken. Because of natural variation in patch size among sampling locations, the length of five parallel transects ranged from 10 m to 31 m. Therefore, the sample grid y-axis and number of samples taken varied among patches. Small y-coordinates were at the *S. patens* end and large y-coordinates were parallel to the tidal creek. The samples collected from the tidal creek edge, denoted by the largest y-coordinate values for each grid transect, were tall-form *S. alterniflora*. All other samples were short-form *S. alterniflora.* We collected entire above-ground *S. alterniflora* stems, stored samples on ice for transport, and transferred samples within 24 h to a −20 °C laboratory freezer for long-term storage.

### Genotyping

Total genomic DNA was extracted from approximately 2 mg of frozen leaf tissue using a Chelex-based protocol modified for *Spartina* spp. from Walsh et al. (1991). Briefly, leaf tissue was cut into ca. 1 cm segments and dropped directly into 1.5 mL safe-lock (Eppendorf) or 2 mL screw top tubes containing four or five 0.125 inch stainless steel ball bearings, 400 µL of Chelex solution (10% w/v BioRad Chelex 100 resin, 50 mM tris-HCl pH 8.0), and 2.0 µL RNase-A (10 mg/mL). Tubes were given one or two 60 s pulses in an MP Biomedicals Fastprep-24 at 4–5 m s^-1^ to homogenize tissue and then incubated for 30 min at 55 °C with occasional vortexing followed by incubation at 100 °C for 8 min. Tubes were twice centrifuged for 2 minutes to pellet solids with the supernatant transferred to a new 1.5 mL tube between spins. DNA was precipitated from the final supernatant by adding 1/10 volume 3M sodium acetate (pH 5.2) and 2x volumes of ice cold 100% ethanol, incubating on ice for 30 min, and centrifuging at maximum speed for 15 min at 4 °C. Pellets were washed in 70% ice cold ethanol, spun at maximum speed for 5 min at 4 °C before decanting the liquid, and air dried overnight before the DNA was dissolved in 100 µL of TE (10 mM tris-HCl pH 8.0, 1 mM EDTA pH 8.0).

We identified ten highly polymorphic microsatellite genetic marker loci after screening over two dozen of the loci described by Blum et al. (2004) and Sloop et al. (2005) as detailed in Tomasula (2023). Each DNA sample was PCR amplified for two multiplex primer sets (Spar05, Spar19, Spar34, Spar07, and Spar02, Spar09, Spar16, Spar20, Spar23, Spar26) using 6FAM, VIC, NED and PET dye labeled primers using Qiagen Type-it microsatellite PCR reagents and thermal profile. Both multiplex PCR amplifications from one DNA sample were combined, mixed with GeneScan 600 LIZ size standard in formamide, and fragments separated by capillary electrophoresis with POP 7 polymer in a 50 cm capillary array on an ABI 3500 Genetic Analyzer. Microsatellite alleles for each locus were scored by binning fragments using the microsatellite plug-in for Geneious Prime (version 2022.2.2) with a 3rd order least-squares sizing curve.

### Identification of genets and ramets

To evaluate the power of the ten genetic marker loci to distinguish genotypes, or quantify the chance of observing different individuals with identical multilocus genotypes due to chance, we estimated the probability of exclusion, (1 - *p*_*sex*_(*F*))^*N*^ where *N* was the number of multilocus genotypes compared (Budowle et al., 2000). The RClone package (Arnaud-Haond and Belkhir, 2007) in R was used to estimate the average expected genotype frequency under sexual reproduction adjusted for departure from random mating with the fixation index, *p*_*sex*_(*F*), using the round-robin allele frequencies method and the fixation index estimation options. To estimate *p*_*sex*_(*F*), all missing genotype data were imputed by randomly sampling from observed allele frequencies at each locus in the total population.

To distinguish unique clonal lineages, or genets, based on microsatellite multilocus lineages, we used Genodive v3.06 (Meirmans, 2020) to estimate a threshold genetic distance under a stepwise mutation model, ignoring missing genotype data. Genets identified using a genetic difference threshold have been termed multilocus lineages, or MLLs (Arnaud-Haond et al., 2007). Multilocus genotypes were considered as belonging to the same MLL if they had genetic distances equal or less than this threshold. Thresholding reduces the impacts of fragment scoring ambiguity and errors, null alleles, and very recent mutations that might occur among ramets.

### Genotype frequencies and genetic differentiation among patches

We estimated the departure from Hardy-Weinberg expected genotype frequencies, *G*_*IS*_, using Nei’s gene diversity method (Nei and Chesser, 1983) in Genodive v3.06 (Meirmans, 2020). We used unique MLLs and Bayesian interesting itemset models (IIM) implemented in INEST 2.31 (Chybicki and Burczyk, 2009) to test for contributions of null alleles (n) and missing genotype data (b) and their combination (nb), plus all combinations with non-random mating (nf, fb, and nfb). These models also estimated average individual inbreeding coefficients based on homozygosity. Estimates were made with default settings for MCMC cycles and burn-in. We estimated allele frequency differentiation among the ten patches and for pairs of patches using *G’’*_*ST*_ under an infinite alleles model using Genodive v3.06. *G’’*_*ST*_ is scaled by the maximum value *G*_*ST*_ can take given the total heterozygosity of the population such that the range of *G’’*_*ST*_ is zero to one, and with correction for a small number of populations (Hedrick, 2005; Meirmans and Hedrick, 2011). Patch differentiation was also estimated using a stepwise mutation model measure *R*_*ST*_ (Slatkin, 1995) with a 95% confidence interval for the total population multilocus estimate based on 5,000 bootstrap resampling iterations over patches with replacement and for pairs of patches using 5,000 jackknife iterations over loci implemented in a Matlab script (Brown et al., 2005).

### Isolation by distance within and among patches

Using centroid coordinates for MLLs with two or more ramets within a patch, pairwise Euclidean geographic distances for all MLLs were estimated within each patch. Bruvo’s genetic distances, which employ a stepwise mutation model (Bruvo et al., 2004), were estimated for each pair of MLLs within patches. Isolation by distance within patches was tested using a Mantel test with Spearman’s rank correlation between Bruvo’s genetic distances and Euclidean geographic distances, implemented in R with the vegan package v2.7.2 and using 9,999 permutations to estimate a null distribution. Geographic distances between patches were estimated using the latitude and longitude of each patch and the distHaversine function of R package geosphere v1.5.20. We tested for isolation by distance among patches using the R function glm with a gaussian error distribution and link function to fit linearized pairwise genetic differentiation estimates (e.g. G’’_ST_/(1-G’’_ST_) on distance between each pair of patches (Rousset, 1997).

### Genet richness and distributions of ramets per MLL

Genet richness (number of MLLs) was determined for the total population and each patch, as well as the mean genet richness across patches. Ramets for each MLL were tallied for the total population and within patches. We used a neutral null model for the number of MLLs observed for each ramet count assuming vegetative expansion of ramets resulted from sampling with replacement over time and were therefore Poisson or negative binomial distributed. This follows how family size is modeled in the Wright-Fisher model of genetic drift in finite populations (Hamilton, 2021) or how occurrence count distributions are modeled in ecological data (e.g. Lindén and Mäntyniemi, 2011). Since observations of zero ramets per MLL were not possible, we employed zero-truncated vector generalized linear models (vglm) in the R package VGAM (Yee, 2015). To test for heterogeneity of rate coefficients among patches, we fit vglm models with and without patch as a factor and used a likelihood ratio test (negative of twice the difference in log likelihoods of the two models) where the chi-squared degrees of freedom were the difference in degrees of freedom of the two models. To obtain fitted values of MLL counts for ramets per MLL that could be compared with observed values, we used the VGAM function dgaitdpois to obtain zero-truncated probability density values based on the regression-estimated rate coefficients for each patch. These probability density values were multiplied by the total number of MLLs for a patch to yield predicted MLL counts for each value of ramets per MLL. The Pearson goodness-of-fit measure was used to evaluate model fit and test for overdispersion according to $\chi^{2}=\sum \frac{{\left(O_{i}-E_{i}\right)}^{2}}{E_{i}}$ where O_i_ are observed counts and E_i_ are model fit expected counts and degrees of freedom were the number of ramets per MLL categories minus the number of parameters estimated. These statistical analyses were implemented in scripts executed in R v4.5.2.

### Genet and ramet spatial patterns

To quantify the spatial organization of MLLs and ramets within patches, we employed two spatial pattern metrics that allowed for MLLs observed only once (singletons) as well as multiple ramets, and served to quantify the spatial distributions of ramets of the same MLL. The cohesion index quantified the aggregation of ramets of an MLL while taking into account how many sample points were occupied by ramets of the same MLL. The cohesion index has a range from zero, indicating a single ramet or multiple ramets that are not adjacent, to 100 as ramets of the same MLL become more clumped and make up an increasing proportion of a patch. The interspersion and juxtaposition index measured how intermingled ramets of an MLL were within a patch, with low values near zero indicating singleton MLLs or MLLs with clumped ramets, and values approaching 100 if numerous ramets of an MLL were surrounded by sample points occupied by different MLLs in a checkerboard pattern. Both spatial metrics were estimated using the R package landscapemetrics v2.1.4 (Hesselbarth et al., 2019). Random permutations (1,000 iterations per patch) of x and y sample coordinates within patches, preserving observed MLL frequencies, were used to generate null distributions of both spatial metrics.

## Results

### Identification of genets and ramets

All ten microsatellite loci were highly polymorphic, exhibiting 7-29 alleles per locus and substantial heterozygosity in the total population (Table 1). Within patches, loci were also highly polymorphic but most contained a subset of the alleles found in the total population (Table 1). Over all loci, 1.65% of single locus genotypes were missing. The expected multilocus genotype frequencies adjusted for the fixation index (*p*_*sex*_(*F*)) had a maximum value of 2x10^−9^, making the estimated probability of exclusion ≤1.87x10^−6^ in a sample of 935. Using the distribution of pairwise genetic distances among all multilocus genotypes (Supplementary Figure S1 in supplement), we set the MLL threshold at a stepwise genetic distance of eight. With the threshold, there were 223 MLLs in the total population. Per patch, genet richness ranged from 5 to 45 MLLs, with a mean of 22.4 MLLs. MLLs were nearly non-overlapping among all patches. Only one MLL (MLL 109) was found in two patches (orange and yellow), which were also the two closest patches among the ten sampling locations. The number of MLLs was substantially less than the number of genotypes expected under strict sexual reproduction with random mating (Table 2).

**TABLE 1:** Sample sizes and genetic measures were estimated for each patch and the total population.

Patch	*N*	*k*	H	*G*_*IS*_
Black	111	3–6	0.427	−0.407
Blue	71	4–13	0.658	0.153
Brown	99	4–10	0.620	0.136
Gray	90	4–12	0.617	0.162
Green	104	3–15	0.570	0.289
Orange	84	3–10	0.600	0.103
Pink	55	5–14	0.667	0.162
Purple	87	4–17	0.565	0.256
Red	160	4–14	0.530	0.307
Yellow	74	4–12	0.458	0.347
Total population	935	7–29	0.571	0.185 (0.035–0.336)

Columns give numbers of samples genotyped (*N*), range of alleles per locus (*k*), observed multilocus heterozygosity without correction for null alleles (*H*), and estimates of the multilocus average fixation index (*G*_*IS*_) using Nei’s gene diversity method. The 2.5% and 97.5% quantiles for *G*_*IS*_ in the total population were estimated by jackknifing over loci.

**TABLE 2:** Genetic measures estimated for unique MLLs only without ramets showed fewer MLLs than expected with a null model of sexual random mating (all comparisons *p* < 0.001).

Patch	*N*	MLLs	Expected genotypes	*H*	*G*_*IS*_
Black	111	5	49.2	0.570	0.167
Blue	71	29	71.0	0.637	0.196
Brown	99	9	99.0	0.635	0.213
Gray	90	26	90.0	0.627	0.175
Green	104	45	104.0	0.564	0.294
Orange	84	26	83.2	0.582	0.189
Pink	55	29	55.0	0.644	0.214
Purple	87	20	87.0	0.539	0.354
Red	160	19	159.7	0.522	0.366
Yellow	74	16	74.0	0.500	0.361
Total population	935	223	934.5	0.582	0.257 (0.112–0.399)

Columns are Numbers of samples genotyped (N), number of microsatellite genotype multilocus lineages (MLLs) defined with a genetic distance threshold of eight, number of multilocus genotypes expected based on 5,000 random allele sampling iterations to generate multilocus genotypes, observed multilocus heterozygosity without correction for null alleles (*H*), and estimates of the multilocus average fixation index (*G_IS_*) using Nei’s gene diversity method. All MLL numbers were less than genotypes expected by sexual reproduction (p < 0.001). The 2.5% and 97.5% quantiles for total population *G_IS_*, were estimated by jackknifing over loci.

Over all ten patches, the majority of MLLs exhibited between one and 26 ramets, although the Red patch had two MLLs with 37 and 47 ramets and the Black patch had one MLL with 101 ramets (Table 2). For all ten patches, the number of ramets per MLL was between 1 and 101 with an average of 4.2 (95% CI 3.0–5.3) and a median of 1.0. For those 127 MLLs with two or more ramets, the average ramets per MLL increased to 7.9 (95% CI 5.5–10.3) with a median of 4.0. Table 3 shows the average and median ramets per MLL and for those MLLs with two or more ramets for each patch.

**TABLE 3:** Maximum, mean, and median ramets per MLL for each patch for all 226 MLLs (one MLL was found in two patches) and for only those 127 MLLs with two or more ramets.

Patch	Max ramets	Mean ramets (95% CI)	Median ramets	Mean ≥ 2 ramets (95% CI)	Median ≥2 ramets
Black	101	22.0 (−32.6–77.0)	2.0	36.3 (−103.0–175.5)	6.0
Blue	8	2.5 (1.6–3.3)	1.0	4.0 (2.7–5.3)	3.0
Brown	26	11.03.0–19.0)	6.0	11.0 (3.0–19.0)	6.0
Gray	19	3.5 (1.4–5.5)	1.0	7.4 (2.7–12.1)	3.5
Green	13	2.3 (1.5–3.0)	1.0	4.7 (3.0–6.3)	4.0
Orange	20	3.2 (1.6–4.9)	2.0	5.1 (2.4–7.9)	3.5
Pink	13	1.9 (1.0–2.8)	1.0	3.6 (1.2–6.0)	2.5
Purple	26	4.4 (1.2–7.5)	1.0	8.4 (1.9–15.0)	5.0
Red	47	8.4 (2.3–14.6)	4.0	15.1 (4.4–25.6)	8.5
Yellow	23	4.4 (1.2–7.6)	1.0	8.3 (2.0–14.5)	5.0

### Genotype frequencies and genetic differentiation among patches

Over the total population the MLLs exhibited a deficit of heterozygosity with *G*_*IS*_ of 0.257 (95% CI 0.112–0.399) with individual patch *G*_*IS*_ values between 0.167 and 0.366 as expected with mating among relatives or self-fertilization (Table 2). Estimates of *G*_*IS*_ do not account for deficits of heterozygosity caused by null alleles. All patches showed evidence in Bayesian IIM models that genotype frequencies were impacted by null alleles, the combination of null alleles and non-random mating, or the combination of null alleles, non-random mating, and missing data (Table 4). The multilocus average individual fixation index estimates (*Fi*) were between 0.02 and 0.114 and all greater than zero in six of the ten patches where estimates were obtained. These estimates of *Fi* are adjusted for effects in the model such as null alleles and show homozygote excess indicating mating among relatives. Allele frequency differentiation among the ten patches showed different magnitudes depending on mutation model assumptions. With an infinite alleles model, *G’’*_*ST*_ = 0.281 (95% CI 0.192–0.387) showed genetic differentiation was moderate and patch pairwise estimates were greater than zero (Supplementary Table S1). Under a strict stepwise mutation model, there was slight genetic differentiation in the total population with *R*_*ST*_ = 0.056 (95% CI 0.015–0.075) and all patch pairwise estimates were not different from zero (Supplementary Table S1).

**TABLE 4:** Bayesian models to test MLL-only genotype frequencies for contributions of null alleles (n), missing genotype data (b) and their combination (nb), plus combinations with non-random mating (nb, nf, fb, and nfb).

Patch	Model	DIC	ΔDIC	Fi
Total	n	16,505.9	16.3	​
	b	18,058.7	1,569.0	​
	nf	16,494.3	4.7	​
	nb	16,496.4	6.7	​
	fb	16,774.2	284.6	​
	**nfb**	**16,489.7**	**0.0**	0.02 (0.3–0.047)
Black	**n**	**228.1**	**0.0**	-
	b	231.1	3.0	​
	nf	230.1	2.0	​
	nb	230.2	2.1	​
	fb	231.4	3.3	​
	nfb	230.0	1.9	​
Blue	**n**	**1876.4**	**0.0**	-
	b	1979.9	103.5	​
	nf	1877.9	1.5	​
	nb	1877.2	0.8	​
	fb	1923.3	46.9	​
	nfb	1879.3	2.9	​
Brown	n	592.8	2.5	​
	b	607.4	17.1	​
	**nf**	**590.3**	**0.0**	0.083 (0.001–0.221)
	nb	592.9	2.6	​
	fb	597.9	7.6	​
	nfb	592.1	1.8	​
Gray	**n**	**1,541.5**	**0.0**	-
	b	1,606.8	65.3	​
	nf	1,544.8	3.3	​
	nb	1,543.5	2.0	​
	fb	1,556.3	14.8	​
	nfb	1,543.7	2.2	​
Green	n	2,895.2	0.4	​
	b	3,141.5	246.6	​
	**nf**	**2,894.9**	**0.0**	0.033 (0.001–0.100)
	nb	2,896.0	1.2	​
	fb	2,966.0	71.2	​
	nfb	2,896.4	1.6	​
Orange	n	1,416.7	15.7	​
	b	1,468.6	67.7	​
	nf	1,416.0	15.1	​
	**nb**	**1,400.9**	**0.0**	-
	fb	1,446.5	45.5	​
	nfb	1,401.5	0.6	​
Pink	**n**	**2041.8**	**0.0**	-
	b	2,128.4	86.6	​
	nf	2044.1	2.2	​
	nb	2042.0	0.2	​
	fb	2064.5	22.6	​
	nfb	2043.8	1.9	​
Purple	n	1,415.9	0.6	​
	b	1,540.1	124.8	​
	nf	1,416.3	1.0	​
	nb	1,416.5	1.3	​
	fb	1,439.1	23.9	​
	**nfb**	**1,415.2**	**0.0**	0.062 (0.001–0.221)
Red	n	1,261.8	8.2	​
	b	1,346.3	92.7	​
	**nf**	**1,253.6**	**0.0**	0.114 (0.012–0.274)
	nb	1,261.6	8.0	​
	fb	1,267.1	13.6	​
	nfb	1,256.1	2.5	​
Yellow	n	1,014.4	0.5	​
	b	1,103.1	89.2	​
	**nf**	1,013.9	0.0	0.102 (0.001–0.262)
	nb	1,016.1	2.2	​
	fb	1,034.3	20.4	​
	nfb	1,015.4	1.5	​

ΔDIC compares the difference in deviance information criterion (DIC) between the best fit model and a given model.The best fitting model is in bold. The multilocus average individual fixation index, Fi, with 2.5% and 97.5% quantiles, is corrected for the variables in the best fit model (dashes indicate no estimate).

### Isolation by distance within and among patches

**FIGURE 2:**
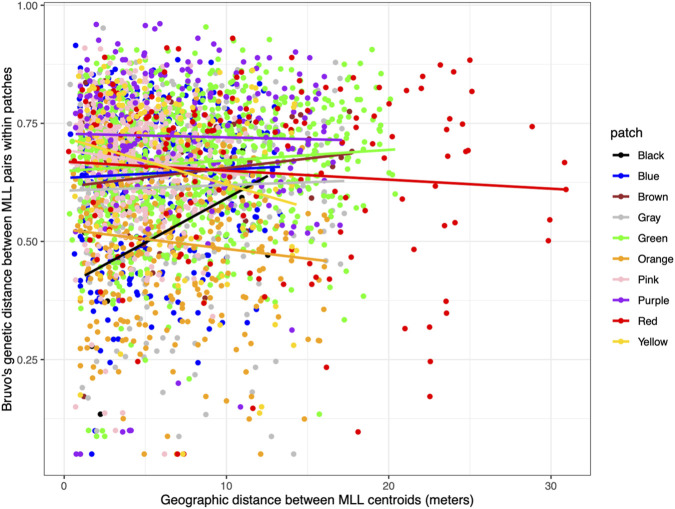
No isolation by distance among pairs of MLLs within patches. Bruvo’s genetic distances and geographic distances between MLLs within patches were used to test for isolation by distance within patches. Geographic distances were estimated using centroid coordinates of all MLLs with two or more ramets. The regression lines are shown here to illustrate trends in patches while Mantel tests showed no correlations between genetic distances and Euclidean distances.

**FIGURE 3:**
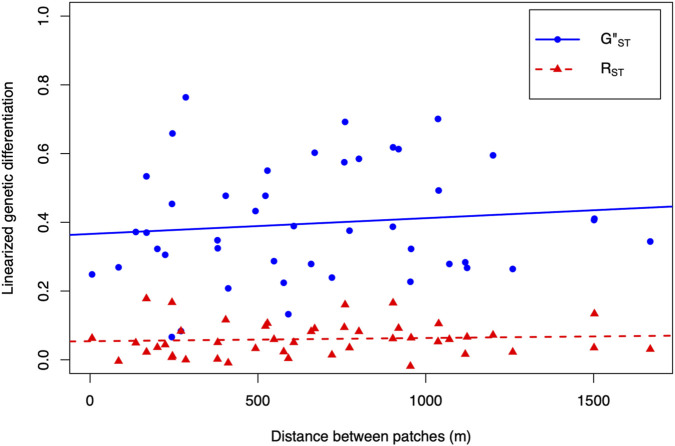
No isolation by distance patterns across patch sampling locations. Genetic differentiation estimates and geographic distances for all unordered pairs of patches to test for isolation by distance. *R*_*ST*_ estimates of genetic differentiation assume a strict stepwise mutation model while *G’’*_*ST*_ estimates are scaled by the maximum *G*_*ST*_ can take given subpopulation heterozygosity, are adjusted for small sample sizes, and assume an infinite alleles mutation model. Genetic differentiation estimates were made using MLLs only without ramets. Geographic distances were estimated using the haversine formula. Neither line had a slope different from zero.

Within patches, there was a wide range of genetic distances between MLLs and no pattern of increasing genetic distance with increasing geographic distance (Figure 2). Mantel tests showed no correlation between pairwise genetic distances of MLLs and Euclidean geographic distances within patches (Supplementary Table S2). Tested at the larger spatial scale of 250–1,000 m between patches, regression models showed that genetic differentiation measured as *G’’*_*ST*_ (coefficient 0.000046, *p* = 0.46) or *R*_*ST*_ (coefficient 0.0.0000092, *p* = 0.62) did not change with increasing geographic distance (Figure 3).

### Distributions of ramets per MLL

**FIGURE 4:**
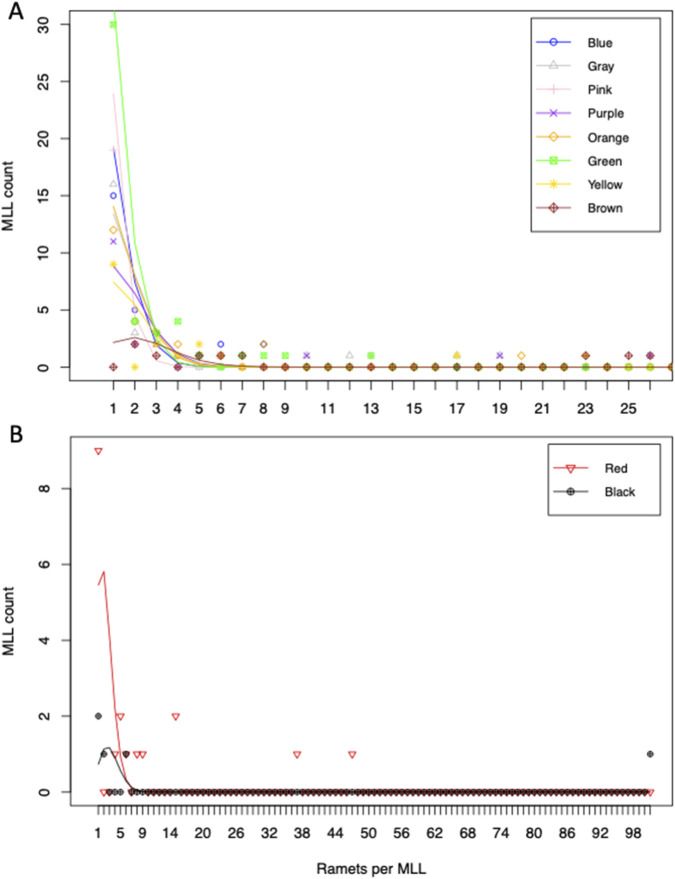
Numbers of ramets per MLL fitted to zero-truncated Poisson distributions to model ramets per MLL as expected by random sampling with replacement. Distributions of the number of ramets per MLL in eight patches with more similar distributions **(A)** and for the two patches that showed three MLLs with very large ramet counts **(B)**. Points are observed counts and lines (without points) connect expected values based on zero-truncated Poisson regression estimated rates.

A zero-truncated Poisson regression model (Table 5) with different rates for each patch (log-likelihood −878.763 with 216 df) was a better fit to ramets per MLL than a model with a single rate for all patches (log-likelihood −1,117.092 with 225 df; likelihood ratio = 3,991.71 with 9 df has *p* < 0.001). The model showed over-dispersion based on the Pearson goodness-of-fit values (≥673,000) for all patches. The zero-truncated Poisson regression was fit to ramets per MLL counts without the 37 and 47 ramet MLLs in the Red patch, and without 101 ramet MLL in the Black patch. Without these three MLLs, the estimated rate coefficients were 0.803 (standard error of 0.370) in the Black patch and 1.485 (standard error of 0.118) in the Red patch. Like the full data set, the data set without the three highest ramet counts showed a better fit for different rates among patches compared to a single rate for all patches (likelihood ratio 181.46 with 9 df has *p* < 0.001). Figure 4 shows observed and zero-truncated Poisson model fitted values of ramets per MLL plotted together for the eight patches with 26 or fewer ramets per genet, and the two patches with MLLs having 31 or more ramets plotted together.

**TABLE 5:** Zero-truncated Poisson regression model coefficients for the number of MLLs predicted by the independent variable number of ramets per MLL.

Patch	Estimated coefficients full data	Coefficient SE full data	Estimated coefficients <31 ramets	Coefficient SE < 31 ramets
Black	3.100	0.095	0.803	0.370
Blue	0.774	0.140	​	​
Brown	2.398	0.101	​	​
Gray	1.206	0.113	​	​
Green	0.674	0.118	​	​
Orange	1.126	0.118	​	​
Pink	0.374	0.181	​	​
Purple	1.456	0.111	​	​
Red	2.131	0.118	1.485	0.118
Yellow	1.471	0.119	​	​

Models with a rate coefficient for each patch were a better fit than models with a shared rate coefficient for all patches. Regressions were fit to the full data set and without three MLLs with large numbers of ramets in the Black (101 ramets) and Red (47 and 31 ramets) patches. The coefficient and standard error (SE) estimates are on a log scale.

A zero-truncated negative binomial regression was fit to the ramets per MLL counts as a possible alternative model given the over-dispersion for the zero-truncated Poisson model. The zero-truncated negative binomial model with single rates for all patches yielded coefficient estimates very close to zero (−13.36 and −16.84 on a natural log scale), while the model for different rates among patches was numerically unstable. Negative binomial models often cannot be reliably estimated when the counts are multi-modal with positive counts followed by one or more zero counts and then additional positive counts, and when most observations are clustered around the value of one (https://cran.r-project.org/web/packages/VGAM/VGAM.pdf). Both of these patterns are seen in the ramet per MLL count data here.

### Genet and ramet spatial patterns

**FIGURE 5:**
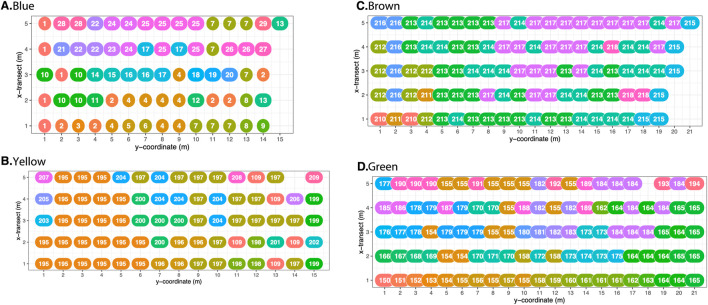
Four representative patches from the ten patches sampled: **(A)** Blue, **(B)** Yellow, **(C)** Brown, and **(D)** Green. Arbitrary numbers and colors designate MLL assignments. All patch sampling grids were oriented with low y-coordinate numbers at higher elevations near a border with *S. patens* and high y-coordinate numbers toward lower elevations near tidal creeks. Shared numbers/colors are vegetative ramets of the same MLL genet. Open grid spaces are missing genotype samples or were marsh areas lacking *S. alterniflora*. See Supplementary Figure S2 for MLL plots of the six patches not shown.

**FIGURE 6:**
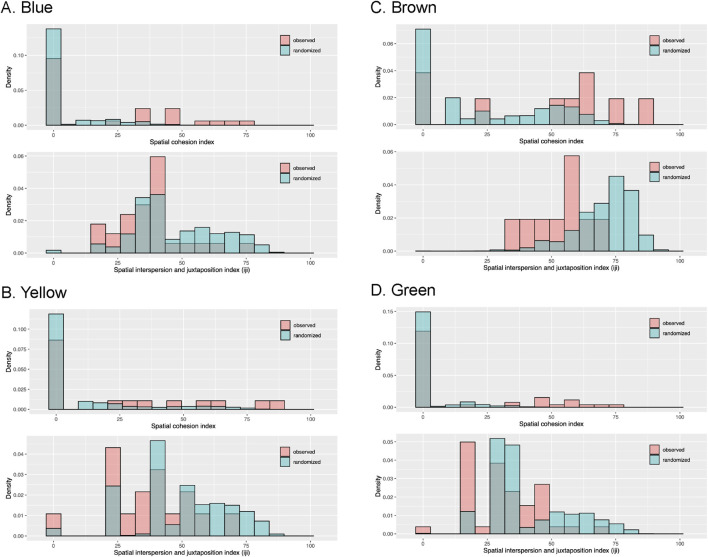
Observed spatial cohesion was higher and spatial interspersion was lower than a random spatial distribution of genets and ramets. Spatial patterns of ramets observed in four of ten patches (**(A)** Blue, **(B)** Yellow, **(C)** Brown and **(D)** Green) summarized with the cohesion index (CI) and the interspersion and juxtaposition index (IJI). The CI measures aggregation of ramets of an MLL while taking into account how many sample points are occupied by ramets of the same MLL, where zero indicates a single ramet or multiple ramets that are not adjacent, and approaches 100 as ramets of the same MLL become more clumped and fill more of a patch. The IJI measures how interspersed an MLL was across a patch, with low values indicating isolated ramet(s) of the same MLL and higher values indicating ramets of an MLL surrounded by sample points occupied by other MLLs or that occupy much of a patch. The null distributions of both spatial metrics were based on 1,000 random permutations of x and y coordinates for each patch. See Supplementary Figure S3 for spatial metric distributions for the six patches not shown.

Maps of four representative patches show genets and their ramets across the sampling grids (Figure 5; maps of the other six patches are in Supplementary Figure S2). Visually, the maps show genets with multiple ramets tended to be aggregated but with variation in the spatial spread of ramets. Assuming that MLLs covered 1 m^2^ around sampling points, MLLs for all ten patches covered between 1 and 101 m^2^ with an average area of 4.2 m^2^ (95% CI 3.0–5.3) and a median area of 1.0 m^2^. For those 127 MLLs with two or more ramets, the average area increased to 7.9 m^2^ (95% CI 5.5–10.3) with a median area of 4.0 m^2^. The cohesion index and interspersion and juxtaposition index (IJI) spatial metric distributions are shown for four representative patches (Figure 6; distributions for the other six patches are in Supplementary Figure S3). The cohesion index had a high frequency of zero values due to genets observed at a single sample point and distributions shifted toward somewhat greater cohesion compared to randomized spatial locations. The IJI distributions showed a range of values as expected with the wide range of ramets per MLL, and distributions were shifted toward greater clustering and less dispersion compared to randomized spatial locations.

## Discussion

Combining high-resolution genotyping, explicit evaluation of the probability of exclusion given sample size, and thresholding in MLL assignment reduced systematic bias and genotyping artifacts in identifying ramets and genets. The number of loci employed and many alleles per locus provided substantial power to distinguish genets from ramets, minimizing the risk of underestimating genet richness or of overestimating ramet number per genet, bias that can arise without sufficient genetic marker power. Studies of genet and ramet identity rarely report probabilities of exclusion, and to our knowledge none have evaluated probability of exclusion in relation to the sample size. As a result, earlier estimates of the clonal structure of salt marsh plants, including classic studies that employed few loci or loci with limited allelic diversity (e.g. Silander, 1979), may have systematically underestimated the number of genets and overestimated ramet abundance due to insufficient genetic marker power. More generally, though thresholding represents best practice for partially clonal organisms (Arnaud-Haond et al., 2007), inconsistent application of genetic difference thresholds for MLL assignment has likely contributed to variation in reported clonal metrics across studies. Thresholding is particularly important for microsatellite data, where null alleles and amplification stutter can generate apparent genotypic differences among ramets of the same genet, artifacts that are often more pronounced with dinucleotide repeat loci such as those used here.

Across all patches, *S. alterniflora* showed a pronounced clonal structure consistent with expectations for a partially clonal species. We detected substantial variation in the number of ramets per MLL and in the genet (MLL) richness within patches. At the scale of the total population sampled, only about one-quarter of the unique multilocus genotypes that would be expected in a strictly sexual, randomly mating population were observed, indicating a strong contribution of vegetative reproduction. MLL genets sampled at a single point made up slightly less than half of the samples with the other half of samples composed of MLLs with two or more ramets. Patches and the total population showed a consistent deficit of heterozygosity among the MLLs, attributable in part to null alleles and missing genotype data in some patches, but also to mating among relatives in all patches. *Spartina alterniflora* is self-compatible and variation among clones in seed set after self-pollination has been observed (Daehler, 1998). The observed deficits of heterozygosity in this study are consistent with partial self-fertilization. For comparison, the *G*_*IS*_ values observed were similar to the equilibrium fixation index of 0.33 expected under a constant mixed mating system with 50% outcrossing and 50% selfing (Hamilton, 2021). Although null alleles contributed to the excess homozygosity, meaning the *G*_*IS*_ estimates of mating among relatives are upwardly biased, this effect was explicitly accounted for in IIM estimates which also showed heterozygosity deficits and evidence of mating among relatives. The *G*_*IS*_ estimates of the mating system are similar to microsatellite genetic marker estimates of the fixation index made in Virginia populations of *S. alterniflora* (Walker et al., 2021). In addition, small genetic neighborhoods where relatedness increases over time, such as in partially clonal plants, can contribute to excess homozygosity, as well as small genetic effective population sizes. Taken together, the substantial deficit of multilocus genotypes relative to a sexual null model, combined with evidence for mixed mating and extensive clonal propagation, supports the interpretation that *S. alterniflora* in this salt marsh has a mating portfolio involving both regular vegetative cloning and sexual reproduction, and that sexual reproduction includes mating among relatives and possibly selfing.

The degree of allele frequency differences among patches was slight to moderate, depending on the mutation model of the estimator, but there was a consistent lack of isolation by distance. The stepwise estimator more closely follows observed mutation patterns for microsatellite loci (e.g. Vigouroux et al., 2002), with increasing weight given to alleles that have greater repeat number differences and therefore less chance of recent state change by mutation. In contrast, the infinite alleles model maximizes perceived genetic differences among alleles by disregarding states, including adjacent repeat states that might have recently diverged via mutation and may not be indicative of genetic isolation. Neither estimator of patch genetic differentiation showed evidence of isolation by distance at the scale of genets within patches or between patches across the salt marsh. At a contrasting spatial scale of populations hundreds of km apart, Blum et al. (2007) found evidence of isolation by distance using chloroplast and nuclear microsatellite genetic polymorphism. Isolation by distance is the null model for genetic differences between and among geographic locations (Meirmans, 2012), and its magnitude is a function of the patterns and magnitude of migration and gene flow over space and time, with possible reductions caused by environmental and landscape barriers. Consistent genetic differences among genets found at lower and higher tidal elevations could produce genetic differences associated with patch distances, caused by environmental filtering based on genotype performance differences that lead to habitat-specific adaptation. There was no evidence of any of these genetic isolation effects at the spatial scale of meters within patches nor at the scale of tens and hundreds of meters between patches.

The Poisson distribution served as a useful neutral null model that imagined counts of ramets per genet as resulting from sampling with replacement. The observed variance in ramet counts greatly exceeded that expected under the Poisson distribution. Such overdispersion is common in ecological data and has many possible causes (Lindén and Mäntyniemi, 2011). Ramets per genet is the product of the net birth-death demography of vegetative modules (Harper, 1980) that is potentially shaped by a range of factors in space and over time including environmental heterogeneity, intra- and interspecific competition, habitat disturbances, and genotype-specific rates of vegetative cloning (Van Drunen et al., 2015). Sampling errors can contribute to overdispersion and the finite and arbitrary boundaries of the grid patches here likely contributed to an excess in low counts if unobserved ramets of sampled genets fell outside the sampling boundaries, especially near edges. The sampling unit for Poisson distribution models of ramet counts is the number of MLLs, and sample sizes of MLLs were about one-quarter of the total number of samples genotyped and smaller yet for each patch. Further, the nature of the count distributions precluded fitting zero-truncated negative binomial models that might better estimate count variance. Future simulation work could make improved estimates of expected ramet per genet count distributions under a range of neutral environmental and ecological influences to develop predictions applicable to empirical data and explore adequate sample sizes needed for estimates.

Despite strong environmental and disturbance gradients across each patch, within patches there was a consistent spatial pattern of ramets within MLLs having intermediate levels of clumping and interspersion with other genets and ramets. The observed spatial distributions are consistent with vegetative expansion by rhizomes that produces a degree of localized ramet clustering as a genet expands in its habitat with some stochasticity in direction and distance leading to unevenness of spread, plus the possibility of death of some ramets over time (e.g. Harper, 1980). Ramets of the same MLL were observed to span sizable areas along the environmental gradient characterized by contrasting inundation, salinity, herbivory, and physical disturbance (Bertness, 1991; Ewanchuk and Bertness, 2003; Brewer et al., 1998; Bertness et al., 2014). In salt marshes, environmental filtering and interspecific competition influences species community assemblage, particularly the dominance of *S. alterniflora* below the daily high tide and *S. patens* above daily high tide (Bertness, 1991). Our results suggest that neither environmental filtering nor intraspecific competition influence spatial patterns of *S. alterniflora* genets and ramets. Within this largely neutral framework, the few MLLs with exceptionally high ramet counts may be best interpreted as outcomes of historical contingency. These abundant MLLs were not confined to a particular subzone and spanned heterogeneous environmental conditions. Yet, they are outliers and the possibility remains that these ramets are high performing and adaptive processes influence their abundance. Even so, they were the exception, not the norm, and overall our findings support interpretations based on priority effects and stochastic vegetative spread.

Our study is among the first to resolve fine-scale clonal spatial patterns and spatial genetic structure within a single *S. alterniflora* population where samples were mapped to environmental heterogeneity, while also explicitly linking these patterns to numerous quantitative neutral null models. Previous work has often employed ecological diversity and richness metrics that are useful summaries of patterns (Arnaud-Haond et al., 2007) but are not easily coupled with mechanistic models of the causes of genet and ramet variation. Empirical studies have either mapped clonal structure without evaluating environmental variation or other possible mechanisms causing ramet and genet patterns (e.g. Walker et al., 2021), or examined genotyped stems sampled at meter scales to estimate fine-scale genetic variation, but employed only genet data and genetic models appropriate for sexual reproduction, neglecting clonal ramets (e.g. Hughes and Lotterhos, 2014). This study’s evaluation of genet-ramet patterns in the context of neutral null models leads to important new conclusions. First, previous studies (e.g., Proffitt et al., 2003; Walker et al., 2021) have proposed that clonal spatial patterns could be explained by a few well-adapted dominant clones. However, we found that the distribution of ramets per MLL approximately follows a zero-truncated Poisson distribution, suggesting that clonal structure at meter scales is consistent with random sampling with replacement, and thus with neutral demographic processes and vegetative growth rather than fine-scale local adaptation. Second, while previous studies have suggested natural disturbances may promote genetic diversity in *S. alterniflora* (Edwards et al., 2005; Noto and Hughes, 2020; Walker et al., 2021), and it has been observed that *S. alterniflora* flowers more in response to disturbance (Zerebecki and Hughes, 2013; Li and Pennings, 2017), our study demonstrated that clonal spatial patterns followed neutral predictions. Third, we found that variation in MLLs within one patch is comparable to variation in MLLs found among sites across a region, e.g. across a coastline (Novy et al., 2010; Walker et al., 2021). Generally, genetic diversity studies in partially clonal plants, including *S. alterniflora*, have provided evidence that populations are more genotypically rich than researchers and practitioners anticipated (Travis et al., 2002; Novy et al., 2010; Hughes and Lotterhos, 2014; Walker et al., 2021).

The spatial extent of individual MLLs in our sampling, often covering areas of ten square meters or more, also has important implications for past and future experiments in natural populations of *S. alterniflora*. Sampling for transplantation or experimental treatment plots of a few square meters, a scale common in numerous past empirical studies, may involve few or perhaps only a single genet and thereby confound treatment and limit genet diversity. *S. alterniflora* exhibits distinct tall-form and short-form phenotypes at the creekside and interior marsh, respectively, thus genetic divergence associated with the tidal gradient has been broadly assumed (Gallagher et al., 1988; Hughes and Lotterhos, 2014; Zerebecki et al., 2021; Hanley et al., 2025), though experimental evidence has been contradictory (Valiela et al., 1978; Gallagher et al., 1988). Our study demonstrates that a single MLL often simultaneously experiences multiple environmental conditions, making adaptation to any one condition unlikely. An epigenotyping-by-sequencing study found epigenetic and environmental causes to explain more variation in tall and short-form phenotypic differences than did genotype differences (Mounger et al., 2022). Variation in the number and identity of genets within experimental plots may therefore represent an unmeasured source of variance contributing to outcomes in past field studies, perhaps warranting reconsideration of conclusions.

Beyond experimental design, the extensive spatial footprint of individual *S. alterniflora* clones suggests that marsh ecosystem structure and resilience may be strongly shaped by historical contingency in clonal establishment and spread. Salt marsh habitat restoration and revegetation efforts require knowledge of quantitative patterns of genotypic diversity and genet and ramet patterns for planning and evaluation (e.g. Sperry et al., 2025). Anthropogenic nutrient enrichment from urban and agricultural sources can destabilize salt marshes, contributing to vegetation dieback and erosion (Deegan et al., 2012; Rippel et al., 2021; Tomasula et al., 2023). The phenomenon of salt marsh dieback, which is characterized by widespread *S. alterniflora* mortality and ecological collapse, has been observed to be spatially heterogeneous, with large contiguous areas severely affected while adjacent areas remain relatively intact (Edwards et al., 2005; Alber et al., 2008). This spatial pattern suggests the possibility that clonal structure and intraspecific genetic variation may contribute to differential vulnerability to dieback. Testing this hypothesis will require future studies to combine clonal genet and ramet genetic mapping with experimental manipulations of genetic diversity and environmental stressors known to induce *S. alterniflora* mortality. Overall, characterizing fine-scale clonal spatial patterns provides a critical foundation for understanding how salt marshes may respond to and recover from environmental stressors.

Many foundation species, from corals to intertidal grasses to riparian trees, are partially clonal (Ellstrand and Roose, 1987; Ellison, 2019). Here we provide evidence that the spatial genetic structure of a foundation grass species is shaped by a mating portfolio that includes substantial vegetative cloning, neutral processes, and with the possibility of historical contingency despite its being located in a landscape with strong environmental heterogeneity. Our approach could be replicated at other *S. alterniflora* dominated salt marshes in order to verify the influence of mating portfolio versus the influence of environmental factors on genet-ramet frequencies and their spatial distribution. Recent studies in seagrasses and corals have documented substantial regional variation in clonal diversity and, in some cases, linked spatial genetic patterns to intense disturbances such as cyclones (McMahon et al., 2017; Torres et al., 2020). While the environmental gradient examined at our study site did not indicate adaptive spatial structuring, more extreme disturbances may still alter clonal spatial patterns in foundation species and warrant explicit investigation. Future studies of clonal foundation plants should prioritize rigorous quantification of genetic marker power, including probabilities of exclusion given sample size, and adopt conservative multilocus lineage assignment using genetic distance thresholds, particularly when employing microsatellite markers prone to stutter and null alleles. Extending such approaches to other grass-dominated foundation species, including *S. patens* and related taxa, may reveal previously unrecognized genet and ramet structure with important implications for community dynamics and ecosystem function. Collectively, integrating high-resolution spatial genotyping with experimental and disturbance-based frameworks will be essential for advancing general theory on how clonality shapes the stability and resilience of foundation species under global change.

## Data Availability

The datasets presented in this study can be found in online repositories. The names of the repository/repositories and accession number(s) can be found in the article/Supplementary Material.

## References

[B1] Alber M. Swenson E. M. Adamowicz S. C. Mendelssohn I. A. (2008). Salt marsh dieback: an overview of recent events in the US. Estuar. Coast. Shelf Sci. 80 (1), 1–11. 10.1016/j.ecss.2008.08.009

[B2] Arnaud-Haond S. Belkhir K. (2007). Genclone: a computer program to analyse genotypic data, test for clonality and describe spatial clonal organization. Mol. Ecol. Notes 7 (1), 15–17. 10.1111/j.1471-8286.2006.01522.x

[B3] Arnaud-Haond S. Duarte C. M. Alberto F. Serrão E. A. (2007). Standardizing methods to address clonality in population studies. Mol. Ecol. 16 (24), 5115–5139. 10.1111/j.1365-294X.2007.03535.x 17944846

[B4] Barrett S. C. H. (2015). Influences of clonality on plant sexual reproduction. PNAS 112 (29), 8859–8866. 10.1073/pnas.1501712112 26195747 PMC4517233

[B5] Barrett S. C. H. Harder L. D. (2017). The ecology of mating and its evolutionary consequences in seed plants. Annu. Rev. Ecol. Evol. Syst. 48, 135–157. 10.1146/annurev-ecolsys-110316-023021

[B6] Becheler R. Diekmann O. Hily C. Moalic Y. Arnaud-Haond S. (2010). The concept of population in clonal organisms: mosaics of temporally colonized patches are forming highly diverse meadows of Zostera marina in brittany. Mol. Ecol. 19 (12), 2394–2407. 10.1111/j.1365-294X.2010.04649.x 20465589

[B7] Bertness M. D. (1991). Zonation of *Spartina patens* and *Spartina alterniflora* in a new England salt marsh. Ecology 72 (1), 138–148. 10.2307/1938909

[B8] Bertness M. D. Shumway S. W. (1992). Consumer driven pollen limitation of seed production in marsh grasses. Am. J. Bot. 79 (3), 288–293. 10.1002/j.1537-2197.1992.tb14550.x

[B9] Bertness M. D. Brisson C. P. Bevil M. C. Crotty S. M. (2014). Herbivory drives the spread of salt marsh die-off. PLoS One 9 (3), e92916. 10.1371/journal.pone.0092916 24651837 PMC3961439

[B10] Blum M. J. Sloop C. M. Ayres D. R. Strong D. R. (2004). Characterization of microsatellite loci in *spartina* species (poaceae). Mol. Ecol. Notes 4 (1), 39–42. 10.1046/j.1471-8286.2003.00556.x

[B11] Blum M. J. Jun Bando K. Katz M. Strong D. R. (2007). Geographic structure, genetic diversity and source tracking of *Spartina alterniflora* . J. Biogeogr. 34 (12), 2055–2069. 10.1111/j.1365-2699.2007.01764.x

[B12] Brewer J. S. Levine J. M. Bertness M. D. (1998). Interactive effects of elevation and burial with wrack on plant community structure in some Rhode Island salt marshes. J. Ecol. 86 (1), 125–136. 10.1046/j.1365-2745.1998.00241.x

[B13] Brown K. M. Baltazar G. A. Hamilton M. B. (2005). Reconciling nuclear microsatellite and mitochondrial marker estimates of population structure: breeding population structure of Chesapeake Bay striped bass (*Morone saxatilis*). Heredity 94, 606–615. 10.1038/sj.hdy.6800668 15829986

[B14] Bruvo R. Michiels N. K. D’Souza T. G. Schulenburg H. (2004). A simple method for the calculation of microsatellite genotype distances irrespective of ploidy level. Mol. Ecol. 13 (7), 2101–2106. 10.1111/j.1365-294X.2004.02209.x 15189230

[B15] Budowle B. Chakraborty R. Carmody G. Monson K. L. (2000). Source attribution of a forensic DNA profile. Forensic Sci. Commun. 2 (3). Available online at: https://www.researchgate.net/publication/267683506_Source_Attribution_of_a_Forensic_DNA_Profile_Forensic_Science_Communications_July_2000 (Accessed December 01, 2025).

[B16] Chybicki I. J. Burczyk J. (2009). Simultaneous estimation of null alleles and inbreeding coefficients. J. Hered. 100 (1), 106–113. 10.1093/jhered/esn088 18936113

[B17] Daehler C. C. (1998). Variation in self-fertility and the reproductive advantage of self-fertility for an invading plant (*Spartina alterniflora*). Evol. Ecol. 12, 553–568. 10.1023/A:1006556709662

[B18] Daehler C. C. Strong D. R. (1994). Variable reproductive output among clones of *Spartina alterniflora* (poaceae) invading San Francisco Bay, California: the influence of herbivory, pollination, and establishment site. Am. J. Bot. 81 (3), 307–313. 10.1002/j.1537-2197.1994.tb15448.x

[B19] Deegan L. A. Johnson D. S. Warren R. S. Peterson B. J. Fleeger J. W. Fagherazzi S. (2012). Coastal eutrophication as a driver of salt marsh loss. Nature 490, 388–392. 10.1038/nature11533 23075989

[B20] Doust L. L. (1981). Population dynamics and local specialization in a clonal perennial (*ranunculus repens*): I. The dynamics of ramets in contrasting habitats. J. Ecol. 69 (3), 743–755. 10.2307/2259633

[B21] Edwards K. R. Travis S. E. Proffitt C. E. (2005). Genetic effects of a large-scale *Spartina alterniflora* (smooth cordgrass) dieback and recovery in the northern Gulf of Mexico. Estuaries 28, 204–214. 10.1007/BF02732855

[B22] Ellison A. M. (2019). Foundation species, non-trophic interactions, and the value of being common. iScience 13, 254–268. 10.1016/j.isci.2019.02.020 30870783 PMC6416672

[B23] Ellstrand N. C. Roose M. L. (1987). Patterns of genotypic diversity in clonal plant species. Am. J. Bot. 74 (1), 123–131. 10.1002/j.1537-2197.1987.tb08586.x

[B24] Elsey-Quirk T. Middleton B. A. Proffitt C. E. (2009). Seed flotation and germination of salt marsh plants: the effects of stratification, salinity, and/or inundation regime. Aquat. Bot. 91, 40–46. 10.1016/j.aquabot.2009.02.001

[B25] Ewanchuk P. J. Bertness M. D. (2003). Recovery of a northern new England salt marsh plant community from winter icing. Oecologia 136, 616–626. 10.1007/s00442-003-1303-7 12802675

[B26] Fang X. Subudhi P. K. Venuto B. C. Harrison S. A. (2004). Mode of pollination, pollen germination, and seed set in smooth cordgrass (*Spartina alterniflora*, poaceae). Int. J. Plant Sci. 165 (3), 395–401. 10.1086/382810

[B27] Gallagher J. L. Somers G. F. Grant D. M. Seliskar D. M. (1988). Persistent differences in two forms of *spartina alterniflora*: a common garden experiment. Ecology 69 (4), 1005–1008. 10.2307/1941255

[B28] Hamilton M. B. (2021). Population genetics. 2nd Ed. Hoboken, NJ: Wiley-Blackwell.

[B29] Handel S. N. (1985). The intrusion of clonal growth patterns on plant breeding systems. Am. Nat. 125 (3), 367–384. 10.1086/284348

[B30] Hanley T. C. Gehring C. A. Deckert R. J. Mortazavi B. Richards C. L. Hughes A. R. (2025). Intraspecific variation in plant–fungal interactions across tidal elevation in a salt marsh. New Phytol. 247 (4), 1875–1886. 10.1111/nph.70262 40511620 PMC12267934

[B31] Harada Y. Kawano S. Iwasa Y. (1997). Probability of clonal identity: inferring the relative success of sexual versus clonal reproduction from spatial genetic patterns. J. Ecol. 85 (5), 591–600. 10.2307/2960530

[B32] Harper J. L. (1977). Population biology of plants. London: Academic Press.

[B33] Harper J. L. (1980). Plant demography and ecological theory. Oikos 35 (2), 244–253. 10.2307/3544432

[B34] Hedrick P. W. (2005). A standardized genetic differentiation measure. Evolution 59 (8), 1633–1638. 10.1111/j.0014-3820.2005.tb01814.x 16329237

[B35] Hesselbarth M. H. K. Sciaini M. With K. A. Wiegand K. Nowosad J. (2019). Landscapemetrics: an open-source R tool to calculate landscape metrics. Ecography 42 (10), 1648–1657. 10.1111/ecog.04617

[B36] Honnay O. Bossuyt B. (2005). Prolonged clonal growth: escape route or route to extinction? Oikos 108 (2), 427–432. 10.1111/j.0030-1299.2005.13569.x

[B37] Hubbell S. (2001). The unified neutral theory of biodiversity and biogeography. Princeton: Princeton University Press. 10.1016/j.tree.2011.03.024 21561679

[B38] Hughes A. R. Lotterhos K. E. (2014). Genotypic diversity at multiple spatial scales in the foundation marsh species, *Spartina alterniflora* . Mar. Ecol. Prog. Ser. 497, 105–117. 10.3354/meps10565

[B39] Hughes A. R. Inouye B. D. Johnson M. T. J. Underwood N. Vellend M. (2008). Ecological consequences of genetic diversity. Ecol. Lett. 11 (6), 609–623. 10.1111/j.1461-0248.2008.01179.x 18400018

[B40] Li S. Pennings S. C. (2017). Timing of disturbance affects biomass and flowering of a saltmarsh plant and attack by stem-boring herbivores. Ecosphere 8, e01675. 10.1002/ecs2.1675

[B41] Lindén A. Mäntyniemi S. (2011). Using the negative binomial distribution to model overdispersion in ecological count data. Ecology 92 (7), 1414–1421. 10.1890/10-1831.1 21870615

[B42] Madritch M. D. Greene S. L. Lindroth R. L. (2009). Genetic mosaics of ecosystem functioning across aspen-dominated landscapes. Oecologia 160, 119–127. 10.1007/s00442-009-1283-3 19214586

[B43] Manel S. Schwartz M. K. Luikart G. Taberlet P. (2003). Landscape genetics: combining landscape ecology and population genetics. Trends Ecol. Evol. 18 (4), 189–197. 10.1016/s0169-5347(03)00008-9

[B44] McMahon K. M. Evans R. D. Van Dijk K. J. Hernawan U. Kendrick G. A. Lavery P. S. (2017). Disturbance is an important driver of clonal richness in tropical seagrasses. Front. Plant Sci. 8, 2026. 10.3389/fpls.2017.02026 29259609 PMC5723400

[B45] Meirmans P. G. (2012). The trouble with isolation by distance. Mol. Ecol. 21 (12), 2839–2846. 10.1111/j.1365-294X.2012.05578.x 22574758

[B46] Meirmans P. G. (2020). Genodive version 3.0: easy-to-use software for the analysis of genetic data of diploids and polyploids. Mol. Ecol. Resour. 20 (4), 1126–1131. 10.1111/1755-0998.13145 32061017 PMC7496249

[B47] Meirmans P. G. Hedrick P. W. (2011). Assessing population structure: F(ST) and related measures. Mol. Ecol. Resour. 11 (1), 5–18. 10.1111/j.1755-0998.2010.02927.x 21429096

[B48] Mounger J. M. van Riemsdijk I. Boquete M. T. Wagemaker C. A. M. Fatma S. Robertson M. H. (2022). Genetic and epigenetic differentiation across intertidal gradients in the foundation plant. Spartina Alterniflora. Front. Ecol. Evol. 10, 868826. 10.3389/fevo.2022.868826

[B49] Nei M. Chesser R. K. (1983). Estimation of fixation indices and gene diversities. Ann. Hum. Genet. 47 (3), 253–259. 10.1111/j.1469-1809.1983.tb00993.x 6614868

[B50] Noto A. E. Hughes A. R. (2020). Intraspecific diversity at two trophic levels influences plant–herbivore interactions. Ecosphere 11, e03121. 10.1002/ecs2.3121

[B51] Novy A. Smouse P. E. Hartman J. M. Struwe L. Honig J. Miller C. (2010). Genetic variation of *Spartina alterniflora* in the New York metropolitan area and its relevance for marsh restoration. Wetlands 30, 603–608. 10.1007/s13157-010-0046-6

[B52] Proffitt C. E. Travis S. E. Edwards K. R. (2003). Genotype and elevation influence *Spartina alterniflora* colonization and growth in a created salt marsh. Ecol. Appl. 13 (1), 180–192. 10.1890/1051-0761(2003)013[0180:GAEISA]2.0.CO;2

[B53] Raffard A. Santoul F. Cucherousset J. Blanchet S. (2018). The community and ecosystem consequences of intraspecific diversity: a meta-analysis. Biol. Rev. 94 (2), 648–661. 10.1111/brv.12472 30294844

[B54] Rippel T. M. Tomasula J. Murphy S. M. Wimp G. M. (2021). Global change in marine coastal habitats impacts insect populations and communities. Curr. Opin. Insect Sci. 47, 1–6. 10.1016/j.cois.2021.02.010 33610775

[B55] Rousset F. (1997). Genetic differentiation and estimation of gene flow from F-statistics under isolation by distance. Genetics 145 (4), 1219–1228. 10.1093/genetics/145.4.1219 9093870 PMC1207888

[B56] Silander J. A. (1979). Microevolution and clone structure in *Spartina patens* . Science 203 (4381), 658–660. 10.1126/science.203.4381.658 17813380

[B57] Slatkin M. (1995). A measure of population subdivision based on microsatellite allele frequencies. Genetics 139 (1), 457–462. 10.1093/genetics/139.1.457 7705646 PMC1206343

[B58] Sloop C. M. McGray H. G. Blum M. J. Strong D. R. (2005). Characterization of 24 additional microsatellite loci in *spartina* species (poaceae). Conserv. Genet. 6, 1049–1052. 10.1007/s10592-005-9084-7

[B59] Sperry K. P. Crosby S. C. Bartholet A. E. Hughes A. R. (2025). Revegetation decisions have genetic consequences: actively and passively restored salt marshes display different genetic diversity and composition. Restor. Ecol. 33, e70122. 10.1111/rec.70122

[B61] Tilman D. Isbell F. Cowles J. M. (2014). Biodiversity and ecosystem functioning. Annu. Rev. Ecol. Evol. Syst. 45, 471–493. 10.1146/annurev-ecolsys-120213-091917

[B62] Tomasula J. (2023). Effects of environmental factors on intraspecific genetic diversity of a foundation plant species and the extended ecological consequences. Washington (DC): Georgetown University.

[B63] Tomasula J. Maguire B. Rippel T. M. Lopez E. Perez S. Arab A. (2023). Conditions for collapse: chronic nutrient enrichment increases native insect density linked to salt marsh dieback. Biol. Conserv. 278, 109882. 10.1016/j.biocon.2022.109882

[B65] Torres A. F. Forsman Z. H. Ravago-Gotanco R. (2020). Shifts in coral clonality along a gradient of disturbance: insights on reproduction and dispersal of *Pocillopora acuta* . Mar. Biol. 167, 161. 10.1007/s00227-020-03777-9

[B66] Travis S. E. Proffitt C. E. Lowenfeld R. C. Mitchell T. W. (2002). A comparative assessment of genetic diversity among differently-aged populations of *Spartina alterniflora* on restored versus natural wetlands. Restor. Ecol. 10 (1), 37–42. 10.1046/j.1526-100X.2002.10104.x

[B67] Utomo H. S. Wenefrida I. Materne M. D. Harrison S. A. (2009). Genetic diversity and population genetic structure of saltmarsh *Spartina alterniflora* from four coastal Louisiana basins. Aquat. Bot. 90 (1), 30–36. 10.1016/j.aquabot.2008.05.003

[B68] Valiela I. Teal J. M. Deuser W. G. (1978). The nature of growth forms in the salt-marsh grass Spartina alterniflora. Am. Nat. 112 (985), 461–470. 10.1086/283290

[B69] Vallejo-Marin M. Dorken M. E. Barrett S. C. H. (2010). The ecological and evolutionary consequences of clonality for plant mating. Annu. Rev. Ecol. Evol. Syst. 41, 193–213. 10.1146/annurev.ecolsys.110308.120258

[B70] Van Drunen W. E. van Kleunen M. Dorken M. E. (2015). Consequences of clonality for sexual fitness: clonal expansion enhances fitness under spatially restricted dispersal. PNAS 112 (29), 8929–8936. 10.1073/pnas.1501720112 26195748 PMC4517236

[B71] Vigouroux Y. Jaqueth J. S. Matsuoka Y. Smith O. S. Beavis W. D. Smith J. S. C. (2002). Rate and pattern of mutation at microsatellite loci in maize. Mol. Biol. Evol. 19 (8), 1251–1260. 10.1093/oxfordjournals.molbev.a004186 12140237

[B72] Walker J. B. Bijak A. L. Blum L. (2021). Genetic diversity and clonal structure of *Spartina alterniflora* in a Virginia marsh. Northeast. Nat. 28 (3), 357–370. 10.1656/045.028.0309

[B73] Walsh P. S. Metzger D. A. Higuchi R. (1991). Chelex® 100 as a medium for simple extraction of DNA for PCR-based typing from forensic material. BioTechniques 10 (4), 506–513. 10.2144/000114018 1867860

[B74] Whitham T. G. Young W. P. Martinsen G. D. Catherine A. Schweitzer J. A. Shuster S. M. (2003). Community and ecosystem genetics: a consequence of the extended phenotype. Ecology 84 (3), 559–573. 10.1890/0012-9658(2003)084[0559:CAEGAC]2.0.CO;2

[B75] Wilkinson D. M. (1999). The disturbing history of intermediate disturbance. Oikos 84 (1), 145–147. 10.2307/3546874

[B76] Xiao Y. Zhao H. Yang W. Qing H. Zhou C. Tang J. (2015). Variations in growth, clonal and sexual reproduction of *Spartina alterniflora* responding to changes in clonal integration and sand burial. Clean. Soil Air Water 43 (7), 1100–1106. 10.1002/clen.201300868

[B77] Yee T. W. (2015). “On VGAM family functions,” in Vector generalized linear and additive models with an implementation in R (New York: Springer). 10.1007/978-1-4939-2818-7_18

[B78] Zerebecki R. A. Hughes A. R. (2013). Snail behavioral preference for flowering stems does not impact Spartina alterniflora reproduction. Mar. Ecol. Prog. Ser. 487, 41–54. 10.3354/meps10349

[B79] Zerebecki R. A. Sotka E. E. Hanley T. C. Bell K. L. Gehring C. Nice C. C. (2021). Repeated genetic and adaptive phenotypic divergence across tidal elevation in a foundation plant species. Am. Nat. 198 (5), E152–E169. 10.1086/716512 34648398

