# Supplementary material for: Mating portfolio and neutral mechanisms are primary causes of genet-ramet frequencies and spatial distributions in smooth cordgrass (Spartina alterniflora) along salt marsh tidal gradients
Source: Front Genet. 2026 May 1;17:1810782. doi: 10.3389/fgene.2026.1810782 (PMC13175536; doi:10.3389/fgene.2026.1810782)
Supplement: Supplementary file 1 [file Supplementaryfile1.docx]

Supplementary Material

# Supplementary Figures and Tables

**Supplementary Table S1.** Genetic differentiation for all pairs of patches based on 224 MLLs (a single MLL was found in two patches). Below the diagonal is *G’’_ST_*, a measure of genetic differentiation that is standardized by the average within patch heterozygosity and employing an infinite alleles model. Above the diagonal is *R_ST_* which uses a strict stepwise mutation model and the variances in allelic sizes within and among patches. All *G’’_ST_* estimates and none of the *R_ST_* estimates were different from zero.

Blue Gray Pink Purple Orange Red Green Yellow Brown Black

Blue -- 0.077 0.056 0.076 0.142    0.048 0.089 0.058 0.042 0.011

Gray 0.371 -- 0.084 0.096 0.138 0.034 0.104 0.086 0.035 0.000

Pink 0.218 0.380 -- -0.009 0.151 0.030 0.056 0.022 0.016 0.067

Purple 0.218 0.355 0.172 -- 0.143 0.022 0.047 0.007 0.014 0.076

Orange 0.382 0.409 0.348 0.312 -- 0.118 0.048 0.059    0.060    0.095

Red 0.280 0.273 0.256 0.209 0.291 -- 0.062    0.034    0.023 0.032

Green 0.323 0.323 0.223 0.271 0.245 0.211 -- 0.002 0.004 0.083

Yellow 0.279 0.365 0.270 0.062 0.199 0.289 0.258 -- -0.019 0.050

Brown 0.234 0.244 0.221 0.193 0.244 0.183 0.117 0.185 -- -0.004

Black 0.397 0.433 0.373 0.369 0.330 0.302 0.376 0.412 0.212 --

**Supplementary Table S2.** Spearman’s rank correlation (*rho*) for Bruvo’s genetic distances between MLLs and Euclidean geographic distances between MLLs estimated with Mantel tests using 9999 permutations to estimate a null distribution. Euclidean geographic distances were computed based on centroid coordinates for those MLLs with two or more ramets.

| Patch | *rho* | *p*-value |
| --- | --- | --- |
| Black | 0.212 | 0.392 |
| Blue | 0.015 | 0.397 |
| Brown | 0.014 | 0.464 |
| Gray | 0.031 | 0.298 |
| Green | 0.066 | 0.062 |
| Orange | -0.082 | 0.876 |
| Pink | -0.139 | 0.985 |
| Purple | -0.109 | 0.967 |
| Red | -0.059 | 0.743 |
| Yellow | -0.182 | 0.957 |

## Supplementary Figures


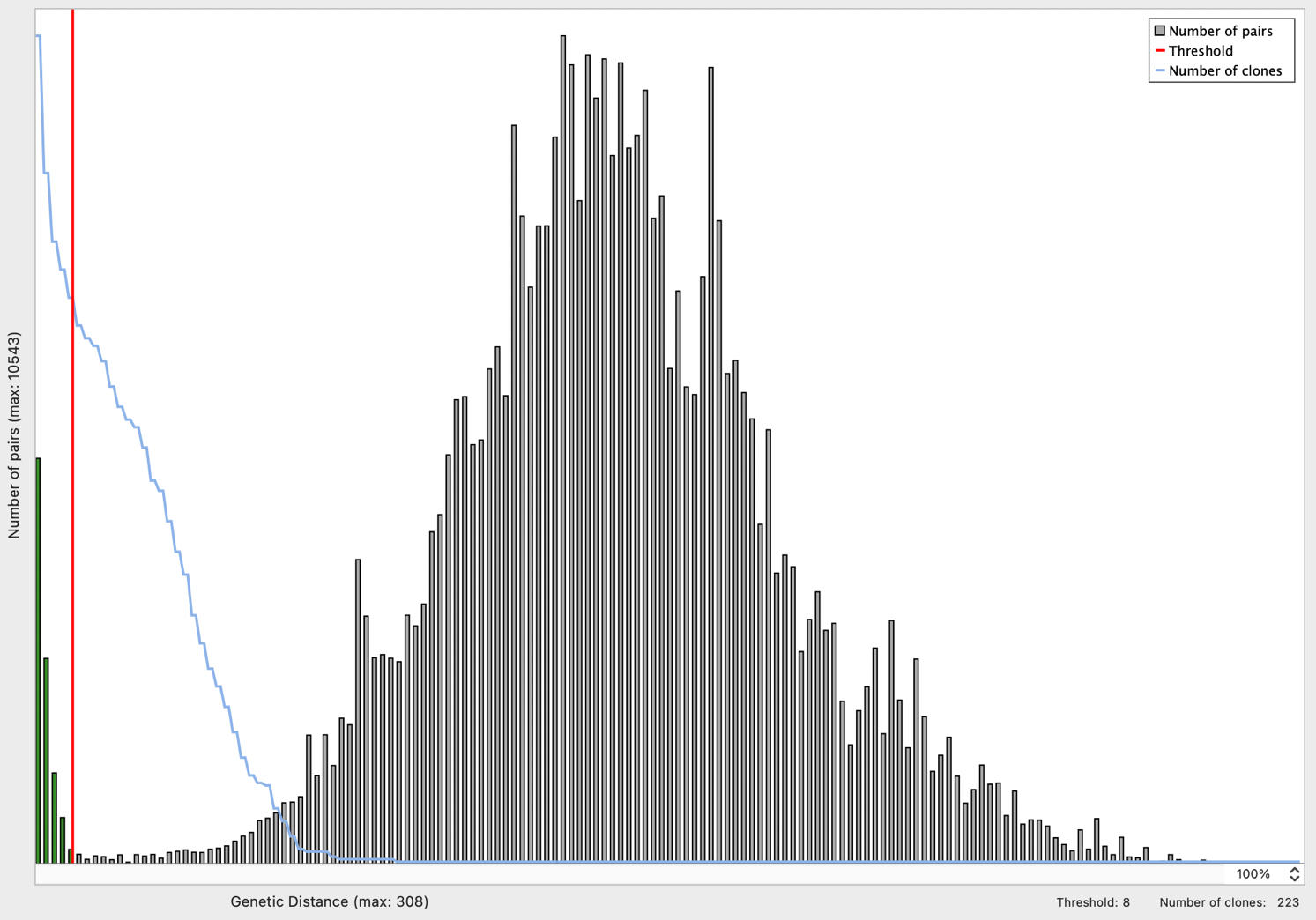


**Supplementary Figure S1.** The distribution of pairwise Bruvo’s stepwise genetic distances for all multilocus genotypes from Genodive. The red vertical line shows a genetic distance of eight, the threshold of genetic distance above which samples were considered distinct multilocus lineages or MLLs.

Black


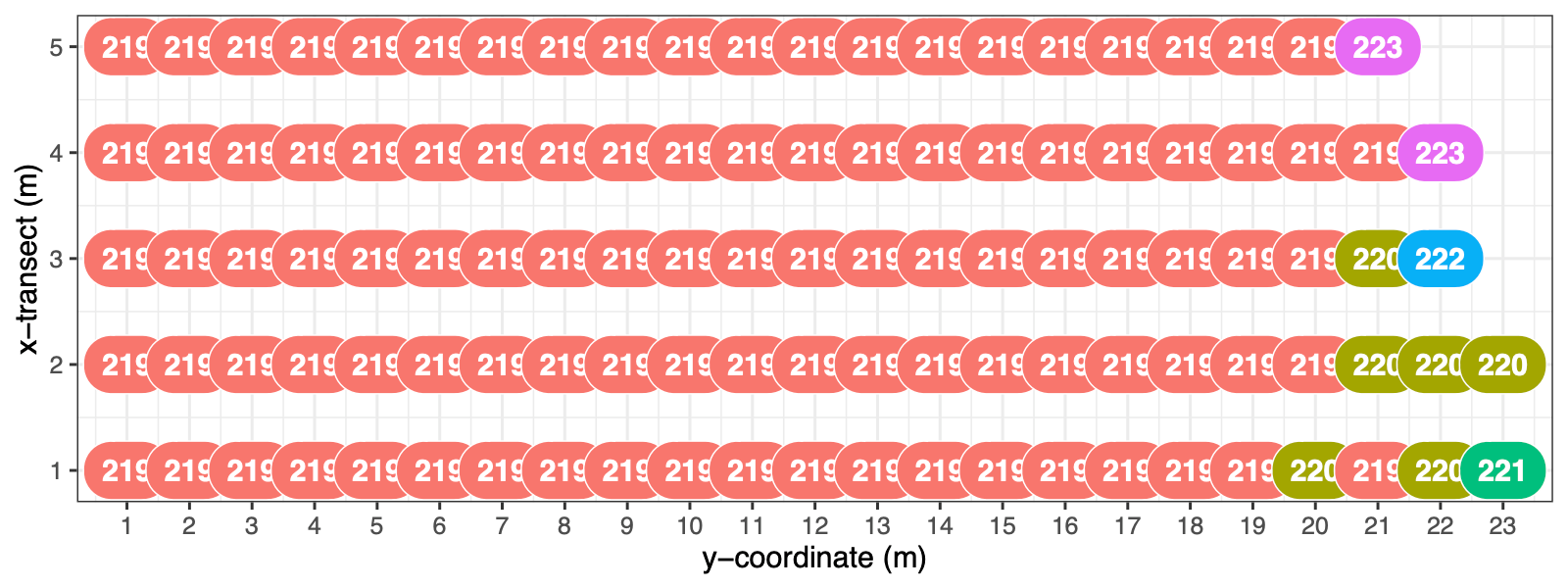


Gray


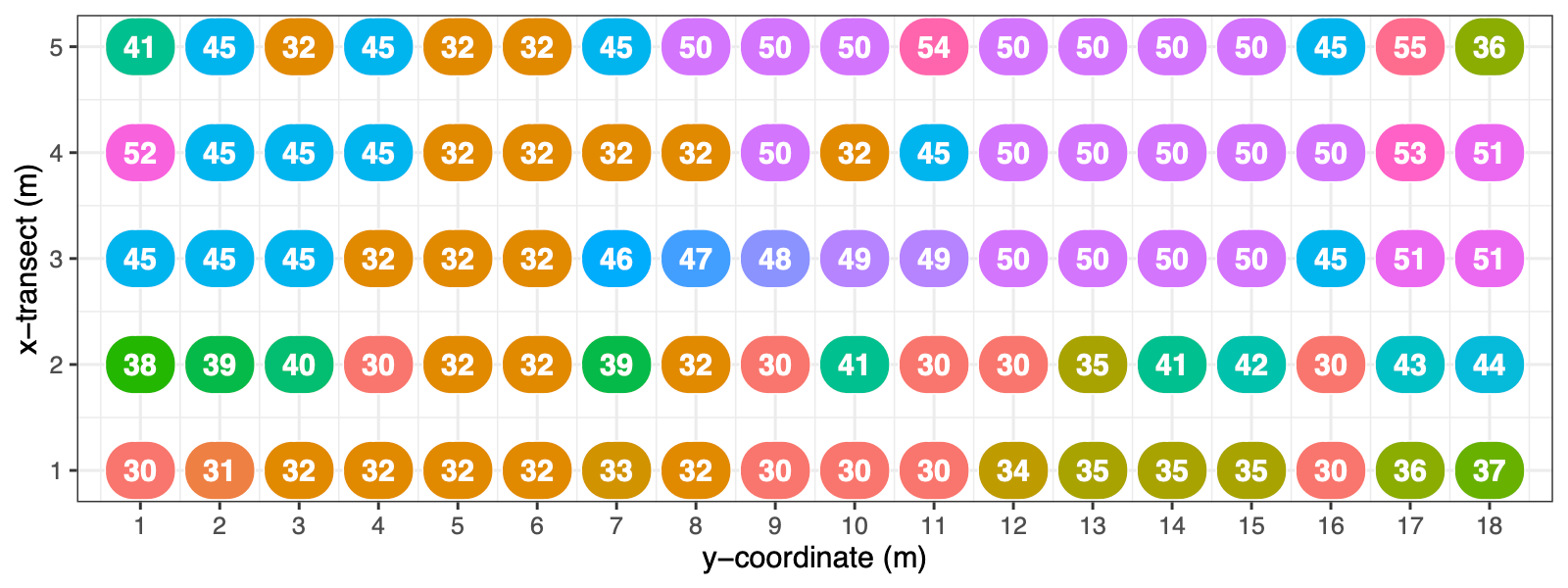


Orange


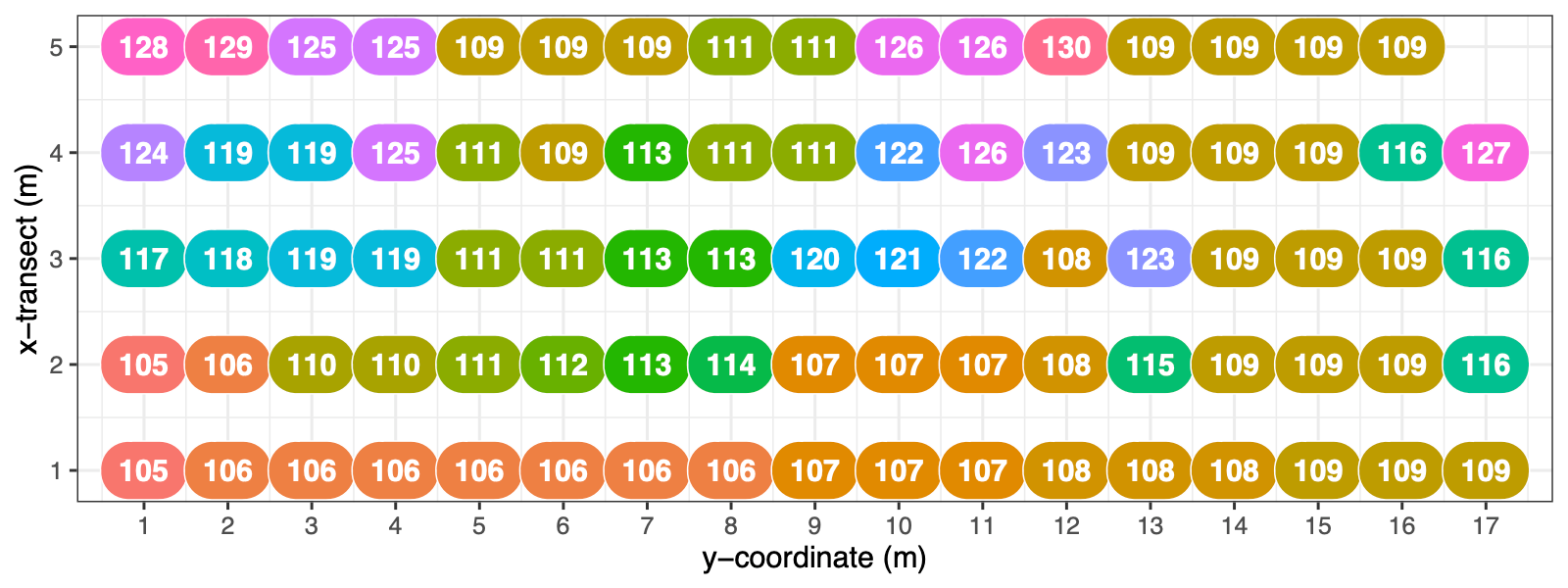


Pink


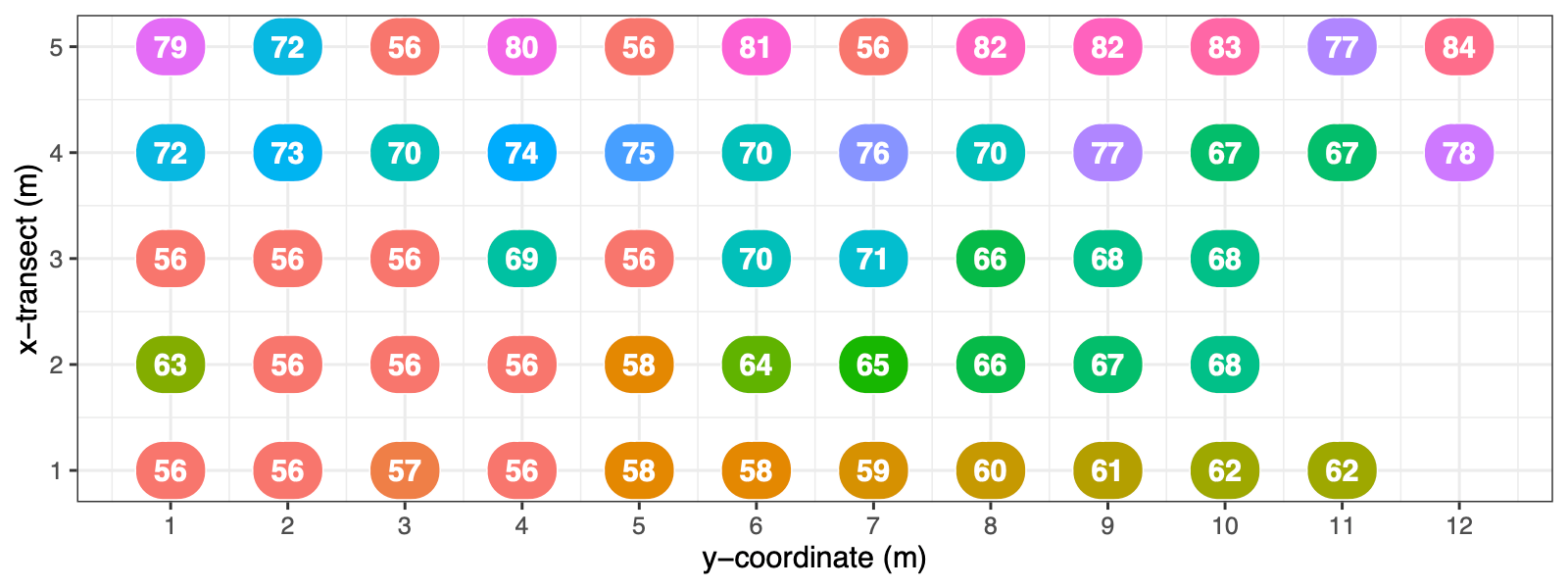


Purple


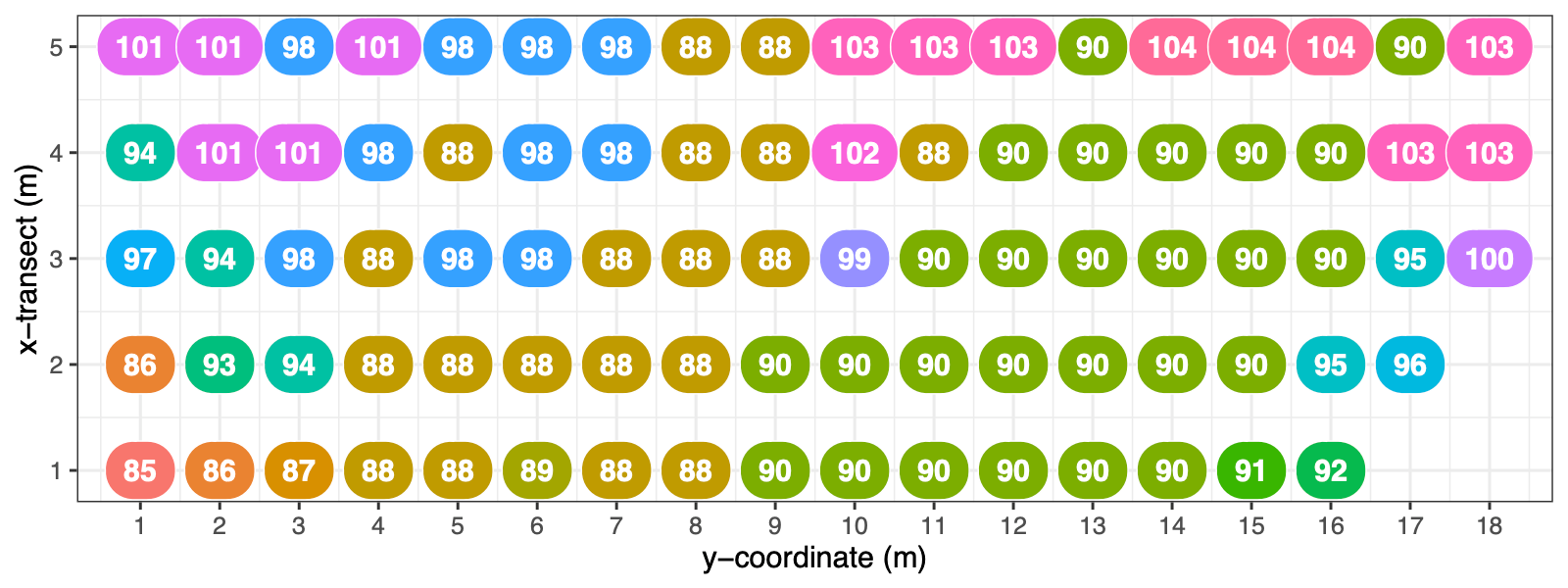


Red


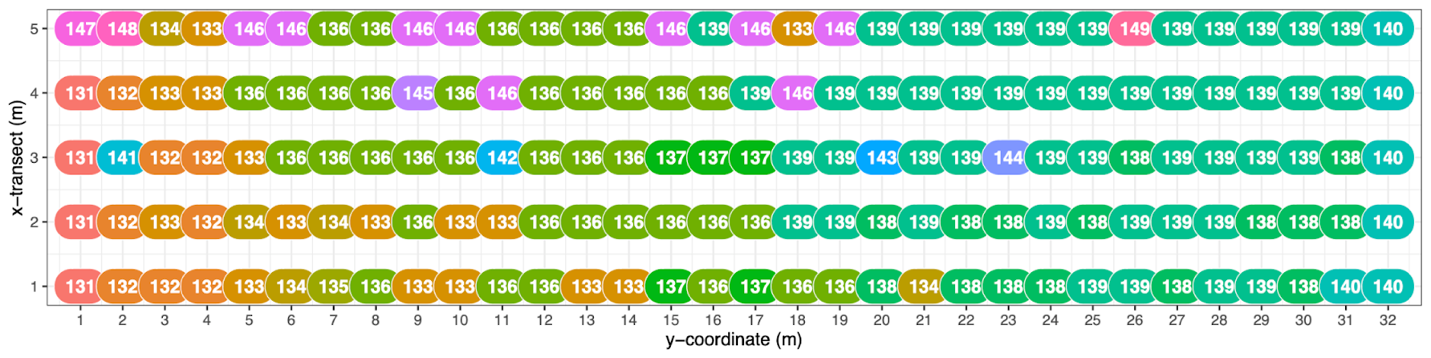


**Supplementary Figure S2.** Six patches from the ten total patches sampled. Arbitrary numbers and colors designate MLL genet assignments. Repeated numbers/colors are vegetative ramets of the same MLL genet within a patch. Open grid spaces are missing samples or areas lacking *S. alterniflora*. The remaining four patches (Blue, Yellow, Brown, and Green) are shown in the manuscript.

Black


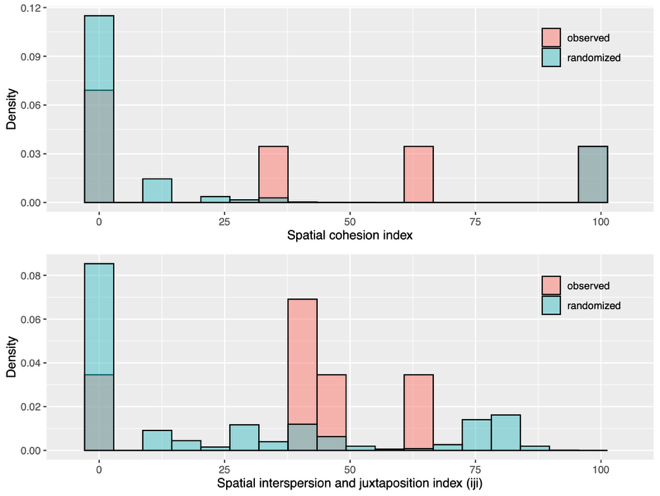


Gray


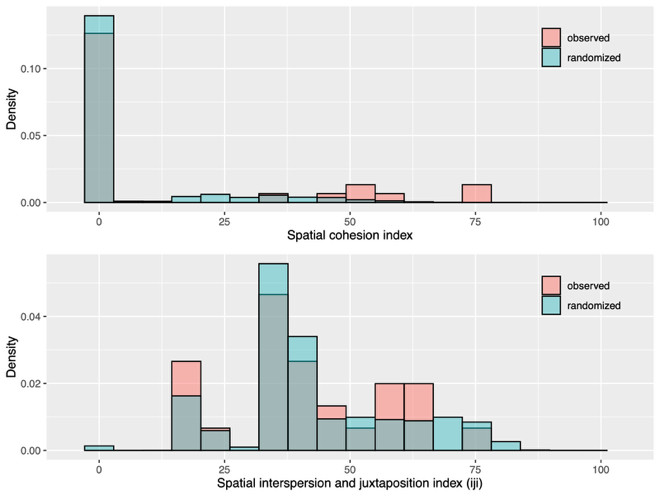


Orange


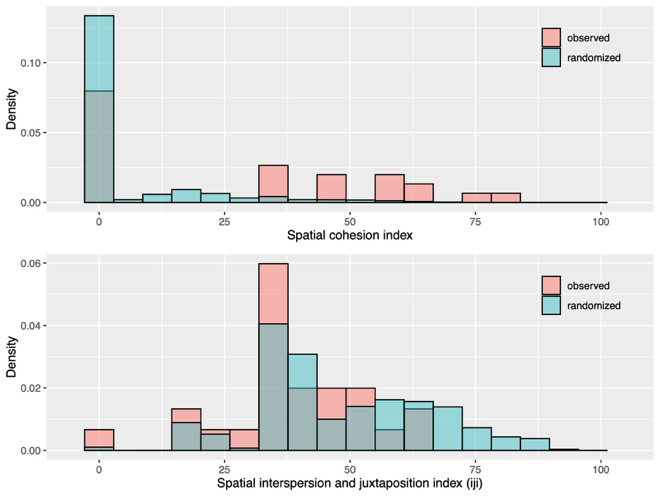


Pink


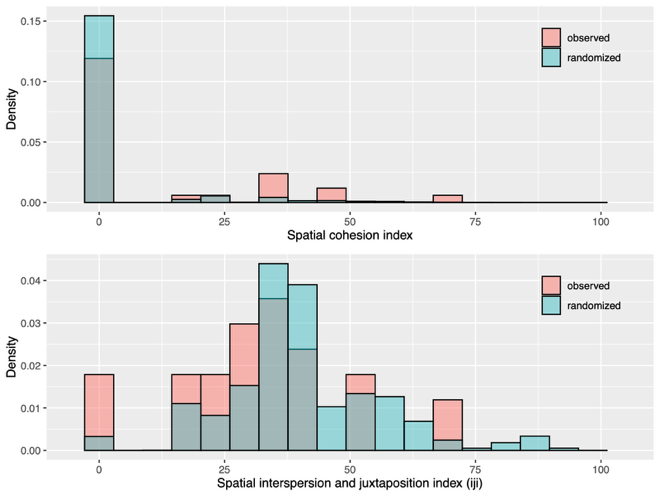


Purple


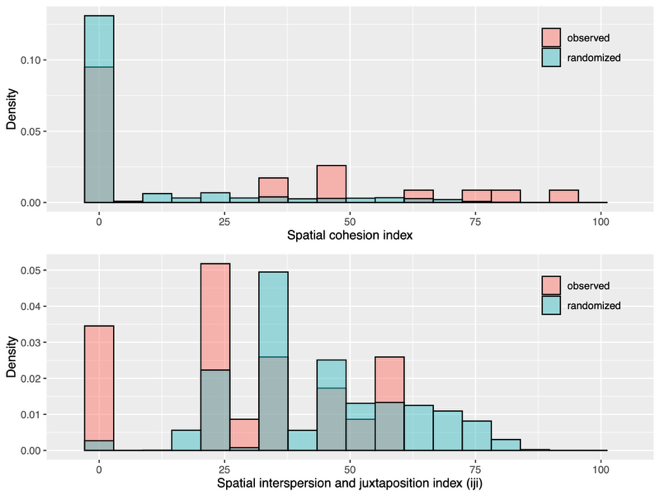


Red


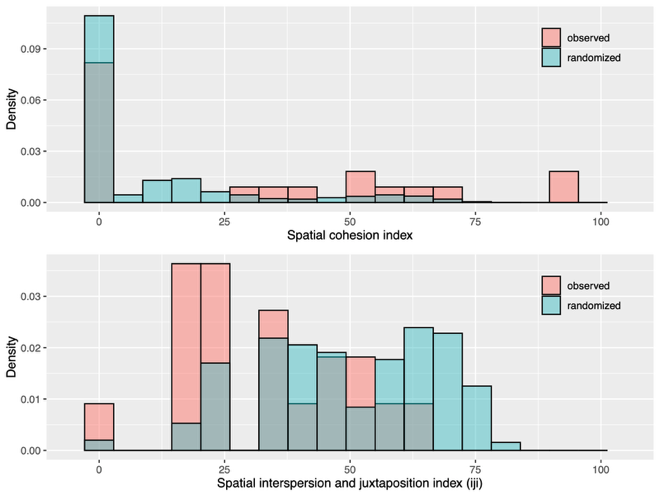


**Supplementary Figure S3.** Spatial patterns of ramets observed within six of ten patches summarized with the cohesion index (CI) and the interspersion and juxtaposition index (IJI). The CI measures aggregation of ramets of an MLL while taking into account how many sample points are occupied by ramets of the same MLL, where zero indicates a single  ramet or multiple ramets that are not adjacent and approaches 100 as ramets of the same MLL become more clumped and filling more of a patch. The IJI measures how interspersed an MLL was across a patch, with a low values indicating an isolated ramet or ramets of the same MLL at the edge of a patch and higher values when ramets of an MLL are found at many sample points or are surrounded by sample points occupied by other MLLs. The null distributions of both spatial metrics were based on 1000 random permutations of x and y coordinates for each patch. The remaining four patches (Blue, Yellow, Brown, and Green) are shown in the manuscript.
